# Enrichment and characterization of a nitric oxide-reducing microbial community in a continuous bioreactor

**DOI:** 10.1038/s41564-023-01425-8

**Published:** 2023-07-10

**Authors:** Paloma Garrido-Amador, Niek Stortenbeker, Hans J. C. T. Wessels, Daan R. Speth, Inmaculada Garcia-Heredia, Boran Kartal

**Affiliations:** 1 Max Planck Institute for Marine Microbiology, Bremen, Germany; 2 Translational Metabolic Laboratory, Department of Laboratory Medicine, Radboud University Medical Center, Nijmegen, The Netherlands; 3 School of Science, Constructor University, Bremen, Germany

**Keywords:** Microbiology, Bacterial physiology

## Abstract

Nitric oxide (NO) is a highly reactive and climate-active molecule and a key intermediate in the microbial nitrogen cycle. Despite its role in the evolution of denitrification and aerobic respiration, high redox potential and capacity to sustain microbial growth, our understanding of NO-reducing microorganisms remains limited due to the absence of NO-reducing microbial cultures obtained directly from the environment using NO as a substrate. Here, using a continuous bioreactor and a constant supply of NO as the sole electron acceptor, we enriched and characterized a microbial community dominated by two previously unknown microorganisms that grow at nanomolar NO concentrations and survive high amounts (>6 µM) of this toxic gas, reducing it to N_2_ with little to non-detectable production of the greenhouse gas nitrous oxide. These results provide insight into the physiology of NO-reducing microorganisms, which have pivotal roles in the control of climate-active gases, waste removal, and evolution of nitrate and oxygen respiration.

## Abstract (web summary)

Multi-omics on a long-term, continuous bioreactor experiment identifies microbes able to respire nitric oxide, a toxic, ozone-depleting, greenhouse gas precursor.

## Main

Nitric oxide (NO) is a strongly oxidizing molecule with important functions in cell biology and atmospheric chemistry. In the atmosphere, NO contributes to air pollution as the precursor of the potent greenhouse gas nitrous oxide (N_2_O), production of acid rain and depletion of the ozone layer^1,2^. In cell biology, NO readily diffuses through cellular membranes and reacts rapidly with other free radicals and with transition metals^3^, making it highly toxic for microbial life^4,5^. The physicochemical properties of NO also make it a valuable signalling molecule^6^ and a key intermediate in the turnover of inorganic nitrogen species^7^, highlighting that microorganisms have evolved strategies not only to detect and detoxify NO but also to respire it very effectively^5,8,9^.

In fact, on early Earth, long before the onset of oxygenic photosynthesis, NO produced through lightning and volcanism was the most powerful oxidant available for life (${E}_{0}^{{{\prime} }}$ = +1.173 V (NO/N_2_O)) (${E}_{0}^{{{\prime} }}$, standard midpoint potential)^10–12^. Consequently, it is hypothesized that, before the emergence of aerobic respiration, NO played a key role in driving the evolution of a bioenergetic pathway related to modern denitrification^12^, in which ancestral NO reductases (NOR) served as the precursors for the terminal oxidases later used in aerobic respiration^13–15^. This suggests that a wide diversity of microorganisms capable of harvesting energy from NO reduction, regardless of its toxicity, must have evolved early in the history of life on our planet.

In the modern nitrogen cycle, NO is a key intermediate in the only two processes that release N_2_ to the atmosphere: anaerobic ammonium oxidation (anammox) and denitrification^7^. During anammox, bacteria from the Planctomycetes phylum reduce nitrite (NO_2_^−^) to NO, which is then used to activate ammonium into hydrazine in the absence of oxygen^16^. In denitrification, NO is turned over during the stepwise reduction of nitrate (NO_3_^−^) to N_2_ (NO_3_^−^ → NO_2_^−^ → NO → N_2_O → N_2_) by a vast diversity of microorganisms that are widespread throughout the tree of life^17^. In contrast to anammox, denitrification can be completed either individually by single microorganisms or, alternatively, by consortia of diverse microorganisms, with each microorganism carrying out one or more of the distinct N-oxide reduction reactions (equations (1)–(4)). In line with this, a variety of N-oxide-reducing microorganisms have been isolated using NO_3_^−^ and the denitrification intermediates NO_2_^−^ and N_2_O, but not NO ^18,19^. However, microbial growth directly on this substrate has been demonstrated; for example, anammox bacteria were shown to grow directly on NO and ammonium in the absence of NO_2_^−^ (ref. ^8^), and denitrifying microorganisms were suggested to increase their biomass when fed with NO under different conditions^20–22^. Still, information about the growth of denitrifying microorganisms on NO is scarce, and our knowledge concerning the physiology of NO reduction, either as a standalone reaction or as part of the denitrification process, is based on cultures that were not obtained using NO and are typically limited to the inhibition and toxic effects of NO on cells^5^.

1 $${\mathrm{NO}}_{3}^{-}+2{\mathrm{e}}^{-}+2{\mathrm{H}}^{+}\to {\mathrm{NO}}_{2}^{-}+{\mathrm{H}}_{2}{\mathrm{O}} \, ({E}_{0}^{{\prime}}=+433 \, {\mathrm{mV}})$$

2 $${\mathrm{NO}}_{2}^{-}+{\mathrm{e}}^{-}+2{\mathrm{H}}^{+}\to {\mathrm{NO}}+{\mathrm{H}}_{2}{\mathrm{O}} \,({E}_{0}^{{{\prime} }}=+360\, {\mathrm{mV}})$$

3 $$2{\mathrm{NO}}+2{\mathrm{e}}^{-}+2{\mathrm{H}}^{+}\to {\mathrm{N}}_{2}{\mathrm{O}}+{\mathrm{H}}_{2}{\mathrm{O}}\,({E}_{0}^{{{\prime} }}=+1{,}175\, {\mathrm{mV}})$$

4 $${\mathrm{N}}_{2}{\mathrm{O}}+2{\mathrm{e}}^{-}+2{\mathrm{H}}^{+}\to {\mathrm{N}}_{2}+{\mathrm{H}}_{2}{\mathrm{O}}\,({E}_{0}^{{{\prime} }}=+1,355\,{\mathrm{mV}})$$

Here we successfully used NO as the direct electron acceptor for the enrichment of NO-reducing microorganisms in a continuous bioreactor. The enrichment culture, mainly comprising two previously unknown members of the betaproteobacterial Sterolibacteriaceae family, ‘*Candidatus* Nitricoxidivorans perseverans’ and ‘*Candidatus* Nitricoxidireducens bremensis’, grew on NO reduction to N_2_ and formate oxidation, with virtually no accumulation of N_2_O. The microbial growth kinetics of the enrichment culture and its affinity for different N-oxides were determined. In parallel, using metagenomics, metatranscriptomics and metaproteomics, the biochemical reactions underlying microbial growth on NO were investigated. This study demonstrates that microorganisms thrive and can be enriched on NO, and presents unexplored opportunities to study microbial turnover on this highly energetic and climate-active molecule that may have been pivotal in the evolution of denitrification and aerobic respiration.

## Results

### Enrichment culture grows with NO as the sole electron acceptor

**Fig. 1:**
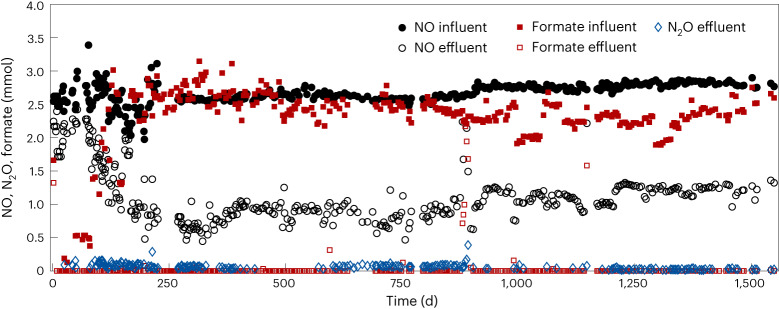
The enrichment culture reduced NO to N_2_ and oxidized formate to CO_2_ in a continuous bioreactor with virtually no accumulation of N_2_O. Values of N_2_O are only shown when the measured concentration was above the detection limit (10–25 ppm). Formate was supplied as the reducing agent for energy conservation and carbon source for biomass growth. Source data

A continuous bioreactor was inoculated with biomass from a municipal wastewater treatment plant (Bremen, Germany) and was continuously fed with NO and formate as the only electron acceptor and electron donor, respectively. The bioreactor was constantly sparged with Ar/CO_2_ to establish anoxic conditions and prevent the chemical conversion of NO with oxygen^8,23^. For 1,549 days, a NO-reducing culture was enriched while converting NO to N_2_ and oxidizing formate to CO_2_ (equation (5)). The activity and growth of the culture was monitored by measuring influent and effluent concentrations of NO, N_2_O, formate and total protein content (Fig. 1 and Supplementary Fig. 1). $\Delta {G}^{0{\prime} }$, Gibbs free energy change under biological standard conditions.

5 $$2{\mathrm{NO}}+2{\mathrm{HCOO}}^{-}+2{\mathrm{H}}^{+}\to {\mathrm{N}}_{2}+2{\mathrm{CO}}_{2}+2{\mathrm{H}}_{2}{\mathrm{O}}\,(\Delta {G}^{0{\prime} }=-367.07\,{\mathrm{kJ}})$$

Cells in the enrichment were predominantly free-living and first retained in the bioreactor using a membrane filter. After 331 days, the culture exhibited constant substrate consumption (Fig. 1) and the membrane was removed. Cells were then washed out at a dilution rate of ~0.104 per day, corresponding to a doubling time of 6.65 days. NO was reduced at a rate of 1.69 (±0.1; *n* = 248) mmol per day (20.93 ± 5.1 µmol per mg protein per day; *n* = 175) and formate was consumed at a rate of 2.38 (±0.2; *n* = 231) mmol per day (29.01 ± 6.3 µmol per mg protein per day; *n* = 165). Because NO was supplied in excess with respect to formate, 700–1,000 ppm of NO always remained in the headspace of the bioreactor, corresponding to 1.5–2.1 µM of dissolved NO (at 20.5 °C). The direct product of NO reduction, N_2_O (equation (3)), was either undetectable (below detection limit (10–25 ppm); *n* = 96) or only detected in the headspace at low concentrations (39.58 ± 20.5 ppm; *n* = 128), indicating that at most ~2.8% of the converted NO could be detected as N_2_O and implying a highly efficient turnover of N_2_O to N_2_ (equation (4)). The observed NO- and N_2_O-reducing activity together with the continuous growth of the culture indicated that the enrichment culture grew on NO reduction to N_2_ coupled to the oxidation of formate.

### Formate is used as an electron donor and organic carbon source

To determine the net biomass yield, we performed a 10-day experiment in the bioreactor in which formate consumption, NO reduction and biomass production were measured. During this period, NO was reduced at 1.57 (±0.02; *n* = 6) mmol NO per day and formate was consumed at 2.44 (±0.06; *n* = 6) mmol formate per day, which is almost identical to the rates observed during routine bioreactor operation. Biomass was produced at a rate of 0.48 (±0.02; *n* = 6) mmol C in the biomass (C-mmol biomass) per day, which corresponded to a net yield of 0.20 (±0.01; *n* = 6) C-mmol biomass per mmol formate.

Overall, during the 10-day experiment, the ratio of NO reduction to formate consumption was 1:1.56 (±0.02; *n* = 6). Based on the expected stoichiometry of NO reduction coupled to formate oxidation (1:1; equation (5)), 64% of the formate consumption could be linked to NO reduction, whereas the assimilation of formate into biomass accounted for a further 20%. The remaining 16% of the consumed formate that could not be linked to NO reduction nor to biomass assimilation is tentatively explained by cell decay and release of dissolved organic carbon, potentially in the form of intermediates and metabolic waste.

### The culture has higher affinity for NO than other N-oxides

Apparent half-saturation coefficients (*K*_m(app)_), maximum reduction rates (*V*_max_) and the substrate inhibition coefficient (*K*_i_*;* for NO only) of the enrichment culture were determined for NO_3_^−^, NO_2_^−^, NO and N_2_O using batch incubations (Table 1 and Supplementary Fig. 2).

**Table 1:** Substrate reduction kinetics of the enrichment culture for NO_3_^−^, NO_2_^−^, NO and N_2_O

	NO_3_^−^		NO_2_^−^		NO			N_2_O	
	*V*_max_	*K*_m(app)_	*V*_max_	*K*_m(app)_	*V*_max_	*K*_m(app)_	*K*_i_	*V*_max_	*K*_m(app)_
Replicate 1	4.59	0.88	9.38	0.55	1.44	0.18	2.05	2.33	0.95
Replicate 2	4.48	0.79	9.16	0.49	1.46	0.15	1.69	2.22	1.05

Results of biological replicate experiments are shown in separate lines. *V*_max_ (µmol N-oxide per mg protein per hour) and *K*_m(app)_ (µM) were both calculated with the Monod equation; *K*_i_ (µM) was calculated with the Haldane equation.

In all experiments, the enrichment culture was immediately active upon substrate addition. Out of the four tested substrates, substrate inhibition was only observed for NO, albeit at a relatively high *K*_i_ of 1.69–2.05 µM. NO was also the substrate for which the enrichment culture showed the highest affinity (*K*_m(app)_ of 153–181 nM). However, compared with the affinity of the few studied denitrifying microorganisms for NO (generally with *K*_m_ values <10 nm)^5,24–26^, the affinity of the enrichment culture was rather low, which was in line with the enrichment conditions of these microorganisms with excess NO.

The affinity values of the enrichment culture for NO_3_^−^, NO_2_^−^ and N_2_O were similar to one another and comparable to those reported for denitrifying and N-oxide-reducing microorganisms^26–33^. The high affinity of the enrichment for N_2_O agreed with the efficient turnover of N_2_O to N_2_ and its absence or transient accumulation in the bioreactor at very low concentrations.

### A new genus dominates the NO-reducing microbial community

To investigate the community composition and metabolic potential of the NO-reducing enrichment culture, total DNA was extracted and sequenced at two different time points (day 497 and day 1,269; Supplementary Table 1). Metagenomic reads were assembled and binned into metagenome-assembled genomes (MAGs) that were annotated and taxonomically classified. From the day 1,269 metagenomic sample, we obtained 12 highly complete MAGs (<3% contamination and >92% completeness) from diverse bacterial phyla (Table 2 and Supplementary Table 2) that represented ~85% of the microbial community in the enrichment culture (as approximated by the fraction of metagenomic reads mapping). Five MAGs contained genes encoding NOR and N_2_O reductases (NOS), which are necessary to reduce NO to N_2_ (equations (3) and (4)). Two MAGs encoded only NOS, suggesting these organisms did not use NO as an electron acceptor and instead reduced N_2_O that might be released by other cells. The five remaining MAGs did not contain any NOR or NOS.

**Table 2:** Characteristics of the MAGs obtained in this study

MAG identifier	Taxonomy	Relative abundance (%)	Presence of respiratory NOR and/or NOS	Completeness (%)	Contamination (%)	Strain heterogeneity (%)	Contigs (*n*)	Genome size (Mbp)	Features (CDS; rRNA; tRNA) (*n*)	Accession number
MAG1 (*Ca*. Nitricoxidivorans perseverans)	Sterolibacteriaceae (f)	63.69	NOR (OHM77_13055), NOS (OHM77_06040, OHM77_06055)	92.28	0.14	0	1^a^	2.7	2,686; 6; 46	CP107246
MAG5 (*Ca*. Nitricoxidireducens bremensis)	Sterolibacteriaceae (f)	12.26	NOR (OEL88_02990), NOS (OEL88_07045, OEL88_07065)	98.18	0.96	0	30	3.6	3,348; 1, 41	JAOTRT000000000
MAG3	Desulfocapsaceae (f)	2.59	–	99.85	2.08	0	27	4.7	4,170; 5; 48	JAOTRX000000000
MAG6	Sterolibacteriaceae (f)	2.47	NOS (OEL86_15165)	97.67	1.29	28.57	41	3.9	3,821; 4; 74	JAOTRU000000000
MAG7	Rhodocyclaceae (f)	0.97	NOR (OEL49_00675), NOS (OEL49_01660, OEL49_10050)	100	0	0	16	3.7	3,415; 0; 51	JAOTRW000000000
MAG8	Rhodospirillales (o)	0.89	NOR (OEL53_08555), NOS (OEL53_02985)	98.26	0	0	57	3.8	3,731; 4; 60	JAOTRY000000000
MAG2	Sterolibacteriaceae (f)	0.62	NOS (OEL20_04110)	97.59	2.41	21.43	69	4.1	3,939; 3; 44	JAOTRV000000000
MAG4	Eubacteriales (o)	0.55	–	98.58	2.13	0	66	3.2	2,957; 3; 43	JAOTSB000000000
MAG9	Ancalomicrobiaceae (f)	0.23	–	98.63	1.58	33.33	236	4.1	3,798; 4; 47	JAOTSD000000000
MAG10	Thiovulaceae (f)	0.22	–	97.96	1.90	50	679	2.1	2,167; 2; 35	JAOTSA000000000
MAG11	Geobacteraceae (f)	0.16	NOR (OEL76_12575), NOS (OEL76_02790)	95.78	1.67	0	161	3.3	3,312; 2; 42	JAOTRZ000000000
MAG12	Bacteria (k)	0.16	–	97.25	0	0	368	3.6	3,000; 3; 44	JAOTSC000000000

The taxonomy of each MAG is shown to the family (f), class (c), order (o) or kingdom (k) level following NCBI taxonomic classification. The relative abundance of each MAG in the metagenome from day 1,269 is expressed as the percentage of metagenomic reads mapping. The locus tags of each NOR and NOS gene sequence from the different MAGs are shown in brackets. CDS, coding sequence; tRNA, transfer RNA.

^a^MAG1 consisted of a single, circular contig that represented a closed genome.

**Fig. 2:**
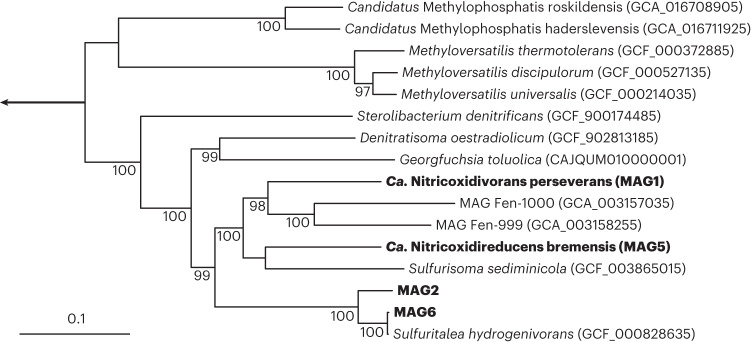
Phylogenetic tree of the Sterolibacteriaceae family based on 120 concatenated single-copy marker genes. Genomes of cultured representatives of the Sterolibacteriaceae family and MAGs from uncultured organisms closely related to *Ca.* Nitricoxidivorans perseverans (MAG1) and *Ca.* Nitricoxidireducens bremensis (MAG5) were obtained from GTDB and NCBI. Organisms obtained in this study are indicated in bold. The genus *Thauera* (GCF_001696715, GCF_002245655, GCF_001051995, GCF_000443165, GCF_001591165, GCF_000310205, GCF_003030465, GCF_001922305, GCF_000310185 and GCF_000310225) was used as the outgroup. The tree was calculated based on maximum likelihood (1,000 iterations) using IQ-TREE. Ultrafast bootstrap values^96^ above 95% are indicated at the branch nodes. The scale bar indicates 0.1 estimated substitutions per site. The set of bacterial single-copy marker genes is according to ref. ^76^.

The two MAGs with the highest relative abundances in the metagenome, MAG1 and MAG5, corresponded to the Sterolibacteriaceae family of the Betaproteobacteria and contained the genes encoding NOR and NOS. The taxonomy of these two MAGs was investigated further by combining whole-genome classification tools with 16S ribosomal RNA (rRNA) identity, average nucleotide identity (ANI; Supplementary Table 3) and average amino acid identity (AAI; Supplementary Table 4) analyses (details described in Supplementary Information). These analyses indicated that MAG1 and MAG5 corresponded to previously unknown genera within the Sterolibacteriaceae family (Fig. 2 and Supplementary Fig. 3). Hence, MAG1 and MAG5 were named *Ca.* Nitricoxidivorans perseverans gen. nov., sp. nov., and *Ca.* Nitricoxidireducens bremensis gen. nov., sp. nov., respectively.

**Fig. 3:**
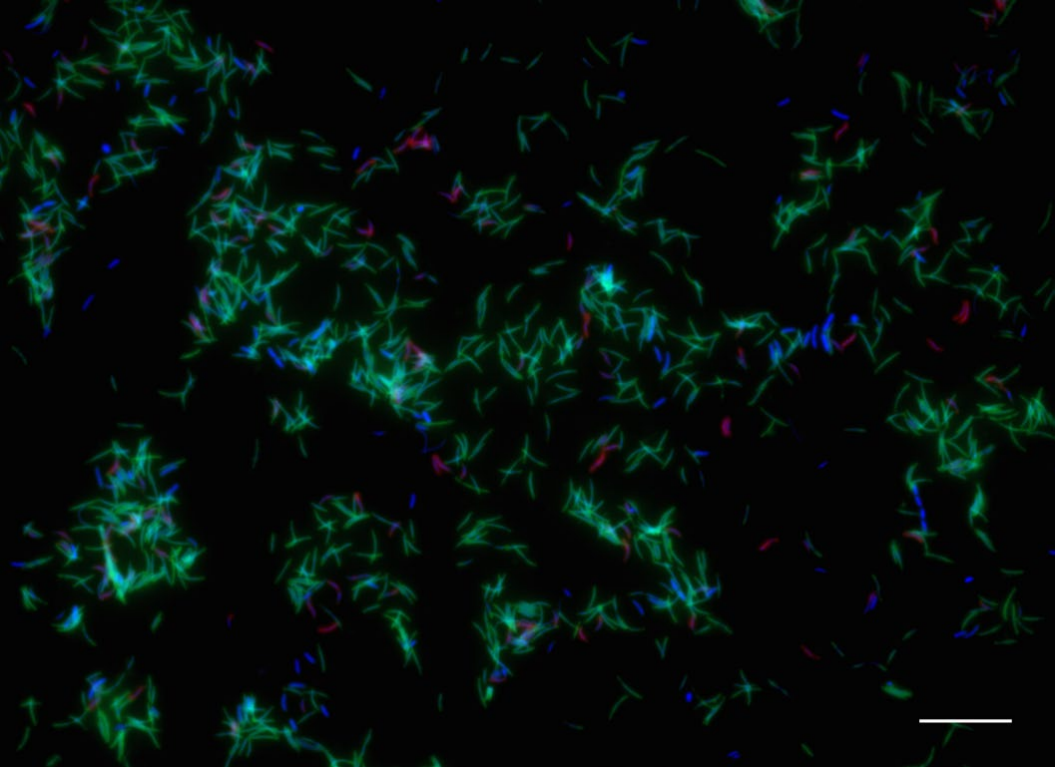
Visualization of *Ca.* Nitricoxidivorans perseverans and *Ca.* Nitricoxidireducens bremensis in the enrichment culture. Double CARD–FISH was performed using probes Nper205 and Nbre448 to target cells of *Ca.* Nitricoxidivorans perseverans (green) and *Ca.* Nitricoxidireducens bremensis (pink), respectively, followed by DAPI staining of all cells (blue). Cell counts were performed in triplicate filter pieces from CARD–FISH and DAPI counts (*n* ≥ 1,000). Scale bar, 10 µm.

The abundance of *Ca*. Nitricoxidivorans perseverans and *Ca*. Nitricoxidireducens bremensis in the inoculum and the enrichment culture was determined using catalysed reporter deposition–fluorescence in situ hybridization (CARD–FISH) with separate oligonucleotide probes. Whereas *Ca*. Nitricoxidivorans perseverans and *Ca*. Nitricoxidireducens bremensis accounted for less than 1% of the population in the wastewater treatment plant (Supplementary Fig. 4), in the enrichment culture, *Ca*. Nitricoxidireducens bremensis constituted 13.66% (±1.6%) of the population and *Ca*. Nitricoxidivorans perseverans became the dominant species with a relative abundance of 81.01% (±3.5%) (Fig. 3).

### All N-oxide reductases are expressed

To investigate the molecular mechanisms underlying the NO- and N_2_O-reducing activity of the enrichment culture, the MAGs of *Ca*. Nitricoxidivorans perseverans and *Ca*. Nitricoxidireducens bremensis, and the metatranscriptome and metaproteome, were scouted for NOR and NOS and associated proteins.

To reduce NO, both *Ca*. Nitricoxidivorans perseverans and *Ca*. Nitricoxidireducens bremensis used a cytochrome *c*-dependent NO reductase (Supplementary Figs. 5 and 6) as they encoded and transcribed catalytic genes *norC* and *norB* and all accessory genes (Table 3). NorB of both organisms and NorC of *Ca*. Nitricoxidivorans perseverans were also retrieved from the metaproteome, along with other proteins encoded in the *nor* gene cluster (Table 3 and Supplementary Tables 5 and 6). The direct product of NO reduction, N_2_O, was reduced by *Ca*. Nitricoxidivorans perseverans and *Ca*. Nitricoxidireducens bremensis using clade II NOS (Supplementary Fig. 7), which often have higher affinity for N_2_O than clade I NOS, in line with the minute concentrations of N_2_O detected in the bioreactor^34–37^. Interestingly, both organisms encoded duplicate copies of *nosZ* genes that were identical to each other (that is, except for the *nosZ* copies in *Ca*. Nitricoxidireducens bremensis that differed in length by one lysine), which, according to our analyses of the Genome Taxonomy Database (GTDB) and Genomes from Earth’s Microbiomes (GEM) databases, is an unusual feature of only ~3.4% of NOS-encoding microorganisms, suggesting the importance N_2_O reduction in *Ca*. Nitricoxidivorans perseverans and *Ca*. Nitricoxidireducens bremensis. All genes from the NOS cluster were detected in the metatranscriptome of both microorganisms (Table 3 and Supplementary Tables 5 and 6). In addition, almost all the NOS proteins from *Ca*. Nitricoxidivorans perseverans were detected in the metaproteome, whereas only the catalytic subunit NosZ from *Ca*. Nitricoxidireducens bremensis could be retrieved, probably due to its much lower abundance in the enrichment culture.

**Table 3:** Genes required for dissimilatory reduction of NO_3_^−^, NO_2_^−^, NO, N_2_O and NO_3_^−^/NO_2_^−^ transporters in the genomes of *Ca.* Nitricoxidivorans perseverans and *Ca.* Nitricoxidireducens bremensis

		*Ca*. Nitricoxidivorans perseverans			*Ca*. Nitricoxidireducens bremensis		
Gene name	Gene product	Locus tag	Abundance in transcriptome (RPKM)	Abundance in proteome (NSAF)	Locus tag	Abundance in transcriptome (RPKM)	Abundance in proteome (NSAF)
NO_3_^−^ reduction							
*narG*	Respiratory NO_3_^−^ reductase catalytic ɑ subunit	OHM77_04925	99.82	6.25 × 10^−5^			
*narH*	Respiratory NO_3_^−^ reductase β subunit	OHM77_04920	130.45	4.82 × 10^−5^			
*narI*	Respiratory NO_3_^−^ reductase γ subunit	OHM77_04910	87.86				
*narJ*	Respiratory NO_3_^−^ reductase molybdenum cofactor assembly chaperone	OHM77_04915	47.66				
*napA*	Periplasmic NO_3_^−^ reductase large subunit	OHM77_07745	88.82	2.68 × 10^−3^	OEL88_03815	242.15	7.60 × 10^−4^
*napB*	Periplasmic NO_3_^−^ reductase small subunit	OHM77_07730	58.20	1.26 × 10^−3^	OEL88_03830	182.77	1.84 × 10^−4^
–	NapC/NirT family cytochrome *c*-type protein	OHM77_07720	108.63	8.62 × 10^−4^	OEL88_03840	246.13	6.63 × 10^−4^
*napD*	Chaperone	OHM77_07750	84.84	1.01 × 10^−4^	OEL88_03810	120.52	
*napG*	Ferredoxin-type protein	OHM77_07740	50.49	6.75 × 10^−5^	OEL88_03820	132.42	
*napH*	Ferredoxin-type protein	OHM77_07735	93.12	2.17 × 10^−5^	OEL88_03825	162.03	
NO_2_^−^ reduction							
*nirC*	*c*-type cytochrome	OHM77_06120	59.86	5.80 × 10^−5^	OEL88_07125	111.64	
*nirF*	Haem *d*_1_ biosynthesis-associated protein	OHM77_06115	66.80	5.95 × 10^−4^	OEL88_07120	119.52	6.16 × 10^−5^
*nirS*	NO_2_^−^ reductase	OHM77_06135	317.67	7.93 × 10^−3^	OEL88_00225	124.31	2.96 × 10^−4^
		OHM77_08200	102.68	2.12 × 10^−4^	OEL88_07140	677.67	7.57 × 10^−4^
NO reduction							
*norB*	NO reductase catalytic subunit	OHM77_13055	101.46	4.02 × 10^−6^	OEL88_02990	161.29	4.01 × 10^−6^
*norC*	NO reductase cytochrome subunit	OHM77_13060	84.90	7.66 × 10^−5^	OEL88_02985	167.17	
*norD*	NO reductase activation protein	OHM77_13030	70.68		OEL88_03015	127.85	1.03 × 10^−5^
*norQ*	NO reductase regulatory protein	OHM77_13040	68.52	2.13 × 10^−5^	OEL88_03005	138.10	
N_2_O reduction							
*nosD*	N_2_O reductase family maturation protein	OHM77_06020	98.68	1.58 × 10^−4^	OEL88_07025	145.25	
*nosF*	Putative ABC transporter ATP-binding NosF protein	OHM77_06005	81.07	4.39 × 10^−4^	OEL88_07010	136.08	
*nosL*	Putative N_2_O reductase accessory protein	OHM77_08590	128.25		OEL88_07005	128.18	
		OHM77_08595	94.11	4.68 × 10^−4^			
*nosZ*	N_2_O reductase	OHM77_06040	302.18	1.19 × 10^−2a^	OEL88_07045	247.59	2.44 × 10^−3^
		OHM77_06055	289.38	1.19 × 10^−2a^	OEL88_07065	240.74	2.44 × 10^−3^
NO_3_^−^/NO_2_^−^ transporters							
*narK*	Putative NO_3_^−^/NO_2_^−^ transporter	OHM77_04930	88.31				
		OHM77_04935	82.37				
*nrtA*	NO_3_^−^/NO_2_^−^ transporter, substrate-binding protein				OEL88_08635	138.69	
*nrtB*	NO_3_^−^/NO_2_^−^ transport system permease				OEL88_08630	148.58	
*nrtD*	NO_3_^−^/NO_2_^−^ transport system, ATP-binding protein				OEL88_08625	135.14	

The abundances of each protein in the metatranscriptome and metaproteome of the enrichment culture are expressed in reads per kilobase per million mapped reads (RPKM) and normalized spectral abundance factor (NSAF), respectively. Duplicate copies of genes *narK*, *nirS* and *nosZ* were present in the MAGs, and their respective abundances are shown in different rows in the table under the same gene name and same gene product.

^a^The abundances of proteins with identical or highly similar sequences in the metaproteome that could not be distinguished between duplicates are shown in this table as the collective abundance of the corresponding protein groups.

The immediate activity of the enrichment culture during substrate affinity assays with NO_3_^−^ and NO_2_^−^ prompted us to explore the metagenome, metatranscriptome and metaproteome for NO_2_^−^ and NO_3_^−^ reductases encoded by *Ca*. Nitricoxidivorans perseverans and *Ca*. Nitricoxidireducens bremensis. Both microorganisms contained two copies of the cytochrome *cd*_1_-type NO_2_^−^ reductase *nirS* and, whereas *Ca*. Nitricoxidivorans perseverans carried both periplasmic (*napAB*) and membrane-bound NO_3_^−^ reductases (*narGHI*), *Ca*. Nitricoxidireducens bremensis encoded only a periplasmic NO_3_^−^ reductase. Furthermore, all these genes, together with the required chaperones and transporters, were detected in the metatranscriptome and, with some exceptions, in the metaproteome (Table 3 and Supplementary Tables 5 and 6).

### Multi-omic analyses reveal one-carbon metabolism

As formate was the only electron donor supplied to the enrichment culture, it was predicted that *Ca*. Nitricoxidivorans perseverans and *Ca*. Nitricoxidireducens bremensis would couple the reduction of NO to formate oxidation. Accordingly, the three subunits of a selenocysteine-containing formate dehydrogenase (FDH) of the N- or O-type were found in the genome of *Ca*. Nitricoxidireducens bremensis (OEL88_12710, OEL88_12715 and OEL88_12720) and in the metatranscriptome and metaproteome of the enrichment culture (Supplementary Table 6). Surprisingly, the genome of the dominant microorganism, *Ca*. Nitricoxidivorans perseverans, did not contain any clearly identifiable FDH. Because its genome was predicted to be closed, based on our analyses, it is unlikely that known FDHs could have been overlooked. Instead, *Ca*. Nitricoxidivorans perseverans could have been using a hitherto unknown FDH or an enzyme with unknown, unconventional formate-oxidizing activity. Alternatively, it might have used other electron donors that were not externally provided to the enrichment culture but made available as metabolic intermediates potentially released by other microorganisms in the enrichment.

To directly assimilate formate as the sole carbon source, formate-tetrahydrofolate ligase (FTL) is required for the first step of every currently known formate-assimilating pathway^38^. Although ~20% of the formate consumed by the enrichment culture had been ascribed to carbon assimilation, genes encoding for FTL could not be found in the genomes of either *Ca*. Nitricoxidivorans perseverans or *Ca*. Nitricoxidireducens bremensis. Other enzymes involved in formate-fixing reactions, such as pyruvate formate-lyase, lactate aldolase and 2-ketobutyrate formate-lyase^38^, were not found either. Formate-consuming microorganisms often use only formate as their energy source and obtain their carbon instead through CO_2_ fixation^39,40^; therefore, it is conceivable that *Ca*. Nitricoxidivorans perseverans and *Ca*. Nitricoxidireducens bremensis used CO_2_ available in the bioreactor for cell carbon assimilation while other microorganisms in the enrichment culture used formate. Indeed, both organisms transcribed all enzymes for the complete Calvin–Benson–Bassham (CBB) cycle (Supplementary Tables 5 and 6) with the exception of sedoheptulose 1,7-bisphosphate aldolase and sedoheptulose 1,7-bisphosphate phosphatase, which were missing from their genomes but whose functions could be performed by fructose 1,6-bisphosphate aldolase and fructose 1,6-bisphosphate phosphatase^41–44^. All the enzymes of the CBB cycle corresponding to *Ca*. Nitricoxidivorans perseverans were also detected in the metaproteome, whereas some of those from *Ca*. Nitricoxidireducens bremensis were missing.

In denitrifying microorganisms, electrons derived from the oxidation of organic or inorganic substrates are transferred to the different N-oxide reductases through an electron transport chain^45^. *Ca*. Nitricoxidivorans perseverans and *Ca*. Nitricoxidireducens bremensis possessed the genes for a NADH–quinone oxidoreductase (complex I), succinate dehydrogenase (complex II), several *c*-type cytochromes, two different haem–copper cytochrome *c* oxidases (including the high-affinity *cbb*_3_ type; complex IV) and an F-type H^+^/Na^+^-transporting ATPase (complex V) (Supplementary Tables 5 and 6). *Ca*. Nitricoxidireducens bremensis also encoded a cytochrome *bc*_1_ complex (complex III), which is used to ultimately transfer the electrons to NIR, NOR and NOS. *Ca*. Nitricoxidivorans perseverans, however, did not contain the genes for either cytochrome *bc*_1_ or its alternatives cytochrome *b*_6_*f* or alternative complex III (ACIII)^46^. We hypothesize *Ca*. Nitricoxidivorans perseverans might bypass complex III altogether and use a different mechanism to transfer electrons from the quinone pool to NOR and NOS, as observed for other denitrifying microorganisms^47,48^.

In addition, *Ca*. Nitricoxidivorans perseverans and *Ca*. Nitricoxidireducens bremensis presented alternative metabolic pathways and the potential for chemolithotrophy through the oxidation of hydrogen and reduced sulfur compounds (Supplementary Information and Supplementary Tables 5 and 6).

## Discussion

In this study, we showed that the cultivation and enrichment of microorganisms directly using exogenous NO as the sole electron acceptor is a successful strategy to obtain NO-reducing microorganisms and study their physiology. Continuous cultivation using excess NO and limiting amounts of formate resulted in the enrichment of two previously unknown species that belong to the relatively understudied Sterolibacteriaceae family, with over 81% of the cells in the culture corresponding to *Ca*. Nitricoxidivorans perseverans and ~14% to *Ca*. Nitricoxidireducens bremensis.

The enrichment culture converted NO to N_2_ with almost no accumulation of the intermediate N_2_O and it could immediately consume NO_3_^−^ and NO_2_^−^, indicating that the culture had the capacity for complete denitrification. Subsequent (meta)omic analyses revealed that both *Ca*. Nitricoxidivorans perseverans and *Ca*. Nitricoxidireducens bremensis used cytochrome *c*-dependent NO reductases (NorBC) and clade II NosZ to reduce NO to N_2_, and had the enzymatic machinery necessary to carry out the complete denitrification process. Furthermore, these organisms expressed NO_3_^−^ and NO_2_^−^ reductases in the absence of these substrates, even when only NO and N_2_O were present. Multiple studies have reported that the transcription of NIR and NOR is co-regulated^49,50^; however, the detection of NO_3_^−^ reductase expression during growth with NO, in the absence of NO_3_^−^, is a rare and interesting observation^47^. In model microorganisms, NO_3_^−^ reductases are believed to be induced by the presence of NO_3_^−^, or low oxygen conditions in the case of NAP^51–53^. Still, it is conceivable that NO might be involved in the regulation of all upstream N-oxide-reducing proteins in a hitherto unappreciated diversity of microorganisms, suggesting that the regulation of N-oxide reduction is more complex than previously assumed.

Of the four N-oxides, the enrichment culture had the highest affinity for NO. Interestingly, at micromolar concentrations of NO that are toxic to other denitrifying microorganisms^54^, the NO-reducing activity of the enrichment culture was only partially inhibited (~40% at 6.6 µM; Supplementary Fig. 2). These observations agreed with the conditions of excess NO that were present in the bioreactor (1.5–2.1 µM) and indicated that the enriched microorganisms not only could grow at nanomolar concentrations of NO but also persisted at high concentrations of this toxic and climate-active gas.

Whereas it was clear that both enriched microorganisms used NO and N_2_O as electron acceptors, unravelling their strategy to obtain reducing equivalents and acquire cell carbon was not straightforward. For over 1,500 days of incubation, ~60% of formate was oxidized by the enrichment culture coupled to NO and N_2_O reduction and ~20% was fixed into the biomass. However, a FDH could not be found in the dominant microorganism *Ca*. Nitricoxidivorans perseverans. It is possible that *Ca*. Nitricoxidireducens bremensis or other microorganisms in the enrichment culture fixed formate into more complex carbon compounds, which were released into the enrichment culture and subsequently oxidized by *Ca*. Nitricoxidivorans perseverans. However, the dominance of *Ca*. Nitricoxidivorans perseverans in the bioreactor suggests that this microorganism directly oxidized formate using a yet-unknown FDH or a protein with previously uncharacterized formate-oxidizing activity, potentially another molybdopterin-containing protein such as one of the highly expressed NO_3_^−^ reductases. Furthermore, based on the absence of key proteins that are required in known formate-assimilating pathways^38^ from the MAGs of *Ca*. Nitricoxidivorans perseverans and *Ca*. Nitricoxidireducens bremensis, neither organism used formate as a carbon source but rather fixed CO_2_ through the CBB pathway. It is likely that *Ca*. Nitricoxidivorans perseverans and *Ca*. Nitricoxidireducens bremensis optimized their cellular activity towards energy conservation by oxidizing most of the available organic electron donors, which are limited in the enrichment culture, and using CO_2_, which is in excess, as a carbon source.

The investigation of just two microorganisms that were enriched using NO allowed us to gain insights into the microbial respiration of this highly reactive gas and the mechanisms used by these organisms to regulate their respiratory chains and acquire cell carbon, some of which did not conform to what has been revealed through studying model organisms, highlighting the limitations of metabolic predictions that are based on genome analyses. Currently, the contribution of microorganisms that are able to grow in a wide range of NO concentrations to nitrogen cycling in natural and engineered environments remains unknown. Nevertheless, it may be speculated that these microorganisms could potentially feed on NO and N_2_O released by other microorganisms, remove nitrosative stress and be involved in minimizing the emission of these climate-active gases to the atmosphere. Such properties might also be key for the potential application of NO- and N_2_O-reducing microorganisms in biological NOx removal from wastewater and waste gas streams. Eventually, probing the physiological and biochemical properties of NO-respiring microorganisms enriched from different environments will allow us to better understand microbial control of NO and N_2_O turnover.

Finally, cultivation and enrichment of even more NO-respiring microorganisms, potentially NO converters that lack one or more of the other N-oxide-reducing enzymes, or those that encode as-yet-unknown NO reductases, will shed further light on the evolution of aerobic and N-oxide respiration pathways and enzymes involved therein, and even uncover biochemical pathways that enable microorganisms to grow using energy gained from NO transformations.

## Methods

### Bioreactor set-up and operation

A continuous bioreactor (working volume 2.4 l; Applikon Biotechnology) was inoculated with biomass (15% v/v) from the nitrogen removal tank of the municipal wastewater treatment plant (hanseWasser Bremen). Immediately after inoculation, NO (10% N25 in He N46; Air Liquide) was introduced to the bioreactor as the sole electron acceptor through the gas phase at a flow rate of 0.3 ml min^−1^ diluted in Ar/CO_2_ (Ar 95% N50, CO_2_ 5% N55; Air Liquide) at a flow rate of 19.7 ml min^−1^ to reach an influent NO concentration of ~2,200 ppm and to keep the bioreactor anoxic. The culture was stirred continuously with two standard turbines at 500 rpm. The bioreactor was kept in a temperature-controlled room at 20 ± 2 °C. The pH of the culture was monitored using a pH electrode (Applikon Biotechnology) and automatically maintained at ~7.3 with 1 M NaHCO_3_. The culture was continuously supplied with mineral medium containing 1 mM ammonium as the nitrogen source and the following nutrients per litre: 150 mg CaCl_2_⋅2H_2_O, 100 mg MgSO_4_⋅7H_2_O, 50 mg KH_2_PO_4_, 0.5 ml Fe solution (10.32 g NTA and 4.72 g FeSO_4_⋅7H_2_O per litre) and 0.5 ml trace element solution (30 g NTA, 207 mg MnCl_2_⋅ 4H_2_O, 115 mg CoCl_2_⋅6H_2_O, 250 mg ZnSO_4_⋅7H_2_O, 1.5 g CuSO_4_, 36 mg NaWO_4_⋅2H_2_O, 50 mg NaMoO_4_⋅2H_2_O, 192 mg NiCl_2_⋅6H_2_O, 28 mg SeO_2_, 150 mg CeCl_3_ and 30 mg H_3_BO_3_ per litre). Formate was added to the influent medium as the sole electron donor, and its concentration in the medium was adjusted according to its consumption in the culture. Formate concentration in the bioreactor was always below the detection limit (<50 µm), with the exception of when disturbances were created in the enrichment culture (for example, bioreactor cleanup). Initially, the medium was supplied at a flow rate of 130 ml per day and biomass was retained in the bioreactor with a custom-made membrane filter (0.2 µm pore size). The formate concentration during this period was gradually increased from 1 mM to 20 mM. On day 331, the membrane filter was removed, the bioreactor was converted to a chemostat and the medium flow rate was increased to 250 ml per day. The formate concentration in the influent medium was then set to 10 mM. The only carbon sources provided for carbon assimilation were formate, bicarbonate in the pH buffer and CO_2_ in the influent gas mixture. The activity and growth of the culture was monitored by sampling twice per week from the liquid culture and the influent medium to determine formate and protein concentrations, and from the influent and effluent gases to measure NO and N_2_O concentrations. Samples for formate measurements were centrifuged for 15 min at 21,000*g*, and supernatants were stored at −20 °C until analysis. Samples for protein measurements were directly stored at −20 °C after sampling. All rate calculations and plots related to the activity of the enrichment culture in the bioreactor were performed using MATLAB R2019a (MathWorks).

### Analytical methods

N_2_O concentrations in the effluent gas stream and, initially, NO concentrations in the influent and effluent gas streams were determined using a 7890B gas chromatography system equipped with a PoraPlot Q column (Agilent Technologies) coupled to a quadrupole GAM 2000 mass spectrometer with a secondary electron multiplier (InProcess Instruments). From day 269 onwards, NO concentrations were determined by online measurements using an nCLD 82 NO_*x*_ analyser (Eco Physics) with a detection limit for NO of 0.1 ppm. NO and N_2_O concentrations in the headspace of batch incubations used during NO and N_2_O affinity assays were measured by injecting gas samples either into the NO_*x*_ analyser or into a 7890A gas chromatography system (Agilent Technologies) equipped with a CP-PoraPlot Q column and a micro-electron capture detector (µECD). Formate concentrations were determined by ion chromatography using a 930 Compact IC flex equipped with a Metrosep A Supp 5–250/4.0 column (Metrohm). Up to day 616, protein concentrations in the enrichment culture were quantified using a protein assay (Bio-Rad), according to the manufacturer’s instructions; after day 616 protein concentrations were quantified spectrophotometrically from lysed cells (1 mM NaOH, incubated for 10 min at 95 °C) using the BCA method (Micro BCA Protein Assay Kit; Thermo Fisher). NO_3_^−^ and NO_2_^−^ concentrations in batch incubations used during NO_3_^−^ and NO_2_^−^ affinity assays were measured following an adapted Griess method^55,56^.

### Determination of kinetic parameters

The affinities of the enrichment culture for NO_3_^−^, NO_2_^−^, NO and N_2_O were determined using batch incubations. For each starting concentration of every substrate, duplicate batch incubations were started with 8–20 ml of biomass collected from the bioreactor. Serum vials of 20ml , 40ml or 60 ml were closed with butyl rubber stoppers and aluminium caps and flushed with Ar/CO_2_ (95:5) to remove air. The biomass was not washed to avoid loss of activity, and was added into the vials either undiluted or after dilution with filter-sterilized spent mineral medium. Inoculated vials were again flushed with Ar/CO_2_ (95:5) and an overpressure of 200 mbar was established. The pH was adjusted to ~7.3 with anoxic 1 M NaHCO_3_. Formate was added in excess as the electron donor for the reduction reactions. NO_2_^−^ and NO_3_^−^ were added to the incubations from anoxic stocks to reach the desired starting concentration (0.5 μM, 1 μM, 3 μM, 5 μM, 7 μM, 10 μM, 15 μM, 25 μM, 35 μM, 45 μM or 60 μM). NO and N_2_O were injected into the headspace from 10% NO (v/v, in He) and 100% N_2_O (N50; Air Liquide) to achieve the desired dissolved concentrations (0.1 μM, 0.2 μM, 0.3 μM, 0.4 μM, 0.6 μM, 0.85 μM, 1 μM, 2 μM, 4 μM, 6.5 μM, 8 μM, 15 μM, 30 μM or 60 μM). After NO and N_2_O additions, vials were incubated for 5–10 min to reach gas equilibration before the first sampling. All vials were incubated at 20 ± 2 °C on a shaker plate at 200–250 rpm. Substrate consumption was monitored by periodically sampling either 0.7 ml of the liquid (for NO_2_^−^ and NO_3_^−^ assays) or 0.1–0.25 ml of the headspace gas (for NO and N_2_O assays) during a maximum incubation time of 2 h or until the substrate could no longer be detected. Samples were withdrawn from the vials after careful flushing of needles and syringes with Ar/CO_2_ to avoid oxygen contamination. The extracted volume was always replaced with the equivalent volume of Ar/CO_2_ (95:5). The liquid samples collected at each time point during the NO_3_^−^ and NO_2_^−^ assays were immediately filtered through 0.22 µm filters and stored at 4 °C until analysis. Headspace gas samples were directly injected into the NO_*x*_ analyser or the µECD gas chromatograph to measure NO or N_2_O concentrations, respectively. After each incubation was finished, samples for protein quantification were collected and stored at −20 °C. The total gas pressure in each vial, pH, and temperature were monitored and recorded to ensure reproducible conditions for all assays and to be used on the calculation of NO and N_2_O solubilities.

The concentrations of NO_3_^−^ and NO_2_^−^ in each sample collected at different time points were directly used to calculate the NO_3_^−^ and NO_2_^−^ reduction rates. Dissolved concentrations of NO and N_2_O were derived from Henry’s law solubility constants and the partial pressures of the gases in the headspace of the vials. The initial substrate concentration in the NO and N_2_O affinity assays corresponded to their aqueous concentration, and the substrate reduction rates were calculated using the total amount of substrate (that is, gaseous and aqueous concentrations of NO or N_2_O in the vials). Substrate reduction rates were normalized by the concentration of protein in each vial. The experimental data was used to describe the Monod kinetics of each substrate following the nonlinear least-squares fitting protocol described elsewhere^57^. As a decrease in NO reduction rates was observed at high concentrations of NO, the Haldane equation was applied to determine the substrate inhibition constant for NO. Goodness-of-fit measures and 95% confidence intervals for the substrate kinetic constants using the two models were calculated using GraphPad Prism 9 (v.9.5.1.; GraphPad Software) and are shown in Supplementary Table 7.

### Biomass yield

To determine the net biomass yield of the enrichment culture (which accounts for both cell growth and decay), the consumption of formate and the production of biomass in the bioreactor were measured every 48 h for 10 days. Liquid samples from the influent and effluent were collected in triplicate every 48 h for formate measurements. Effluent samples were centrifuged for 10 min at 4 °C and 21,000*g*, and both effluent supernatants and influent samples were stored at −20 °C until analysis. Triplicate samples for protein quantification were collected from the effluent and stored at −20 °C until analysis. The elemental composition of the biomass in the bioreactor was determined using a Vario Micro Cube elemental analyser (Elementar Analysensysteme) and used to calculate the concentration of carbon in the biomass (C-mmol biomass). Every 48 h, 5 ml of the enrichment culture was collected in triplicate and filtered through pre-combusted Whatman GF/F 25 mm glass microfiber filters (GE Healthcare Life Sciences) using a 200–400 mbar vacuum. Cells were rinsed on the filters two to three times with phosphate-buffered saline buffer. Filters were dried for 2 h at 60 °C, incubated overnight in a desiccator chamber with 12.5 M HCl and dried again at 60 °C. Whole filters were then packed into tin capsules and measured using the elemental analyser. Blank filters were included in the analysis to account for background carbon and nitrogen. A standard curve with sulfanilamide (C_6_H_8_N_2_O_2_S) was prepared following the same procedure to calculate the amounts of carbon in the samples.

### Metagenome and metatranscriptome sequencing and analysis

On days 497, 1,269 and 1,304, 15–20 ml of biomass was collected and pelleted by centrifugation for 20 min at 4 °C and 7,942*g*. Genomic DNA was extracted from cell pellets using the DNAeasy PowerWater kit (Qiagen) following the manufacturer’s instructions. Total RNA was extracted using the RNAeasy PowerWater kit (Qiagen). The resulting concentrations of DNA and RNA were determined with a Qubit 3.0 fluorometer (Thermo Fisher Scientific). DNA and RNA library preparation and sequencing were performed by the Max Planck-Genome-Centre Cologne (https://mpgc.mpipz.mpg.de/home/) using an Illumina HiSeq 3000 instrument (Illumina) to generate paired-end (2 × 150 bp) reads from DNA and RNA samples, and a PacBio Sequel system (Pacific Biosciences) with 1 SMRT cells to generate long reads from DNA samples. Short reads obtained from DNA sequencing with Illumina were trimmed with Trimmomatic 0.39 (ref. ^58^) and assembled using SPAdes 3.15.3 with the -meta option^59^. Binning of the assembled contigs was performed with Maxbin 2.2.7 and Metabat 2.12.1, and high-quality bins were selected with the DAS_Tool 1.1.1. Long reads obtained from PacBio were assembled with Canu 1.9 (ref. ^60^) and Flye 2.9 (ref. ^61^). The completeness, contamination and strain heterogeneity of each bin was determined using CheckM 1.1.2 (ref. ^62^). Short reads from RNA sequencing were trimmed with Trimmomatic, and any ribosomal RNA present in the transcriptome was removed using SortMeRNA 4.1 (ref. ^63^). The metagenomes were automatically annotated with Prokka 1.14.5 (ref. ^64^), and manual refinement of the annotation of selected genes was performed comparing the annotation of each gene in the NR (GenBank 249; National Center for Biotechnology Information (NCBI)), Pfam (35.0), UniProt (2022_1) and KEGG (101.0) databases. Proteins of interest potentially involved in formate metabolism, but initially missing from MAG1 or MAG5, were further searched for by aligning the sequences of said proteins with those present in members of the Sterolibacteriaceae family using MAFFT 7.407, creating profiles for each protein using hmmbuild (HMMER package 3.3.2) and comparing them using hmmscan (HMMER package 3.3.2). In addition, the protein complement of MAG1 and MAG5 was used as a query in an RPS-BLAST search against the NCBI conserved domain database to further ascertain that no proteins with known formate assimilation activity were overlooked. The presence of genes encoding FDH in MAG1 was investigated further by searching the protein complement of MAG1 against a custom molybdopterin protein database with sequences retrieved from the GTDB (release 207) and GEM (release 2021)^65,66^, and against recently published non-canonical FDHs^67^. The custom database contained all protein sequences from the molybdenum-binding subunits of proteins in the complex iron–sulfur molybdoenzyme (CISM) superfamily, including all known FDHs^68^. Four proteins, corresponding to the large subunits of different molybdoenzymes, were identified: proteins OHM77_07745, OHM77_04925 and OHM77_12545, annotated as periplasmic and membrane-bound NO_3_^−^ reductases (NapA and NarG) and sulfite dehydrogenase (SoeA), respectively, which are well-studied members of the CISM family; and protein OHM77_03730, annotated as a hypothetical protein, which did not resemble any known FDH or other characterized molybdopterin protein. The transcriptome abundance of the genes in MAG1 and MAG5 was determined by mapping the reads on the gene sequences of each MAG using minimap2 (2.26)^69^ and subsequently calculating the read counts per gene, with both steps integrated in coverM (0.6.1; https://github.com/wwood/CoverM). The read counts were then corrected for gene length and the total number of reads in the transcriptome dataset to obtain the RPKM.

To investigate the frequency of multiple copies of *nosZ* in the genomes of denitrifying and N_2_O-reducing microorganisms, a genome dataset consisting of the species representatives of the GTDB and the ‘GEM-OTU’ set of the GEM was dereplicated at 95% ANI using fastANI (1.32)^70^. The final dataset containing 78,768 genomes was searched for the *nosZ* gene using DIAMOND (2.0.15)^71^ with a BLAST score ratio approach as previously described^72^. A total of 6,004 *nosZ* genes were recovered across 5,799 unique genomes, with 195 genomes (3.4 %) containing more than one copy of the gene.

Taxonomic placement of the retrieved MAGs was achieved using CheckM 1.1.2, GTDB-Tk 1.7.0 (ref. ^73^) and MiGA 1.1.2.2 (ref. ^74^). In the case of MAG1 and MAG5, their taxonomic affiliation was complemented with 16S rRNA gene phylogenies and ANI and average AAI analyses. The ANIs between our MAGs and cultured representatives of the Sterolibacteriaceae family were calculated with FastANI 1.33 after treatment with EMBOSS (6.5.0) for the genomes comprising more than one contig to obtain concatenated genomes. The AAI was calculated with the script aai.rb (ENVEOMICS 0.1.1)^75^ using the amino acid sequence of the proteins. A whole-genome tree was constructed using 120 concatenated single-copy marker gene sequences^76^ of MAGs obtained in this study that are related to the Sterolibacteriaceae family (MAG1, MAG5, MAG2 and MAG6), two publicly available MAGs closely related to MAG1 and MAG5, and the genomes of cultured members of the Sterolibacteriaceae family available in GTDB and NCBI. For 16S rRNA gene phylogenetic tree calculations, we used the 16S rRNA gene sequences of the same MAGs obtained in this study and included in the whole-genome tree, those from the Sterolibacteriaceae family available in the SILVA database (SILVA SSU Ref NR 99 138.1)^77^ and NCBI, and, as an outgroup, we selected sequences from the genus *Thauera* of the family Rhodocyclaceae.

To calculate the HCO tree, sequences from each family and the NOR proteins present in cultivated members of the Sterolibacteriaceae family were retrieved using blastp against NR (GenBank 249; NCBI). For NosZ tree calculations, we used proteins of the different clades available in the FunGene repository (7.3)^78^ and the NosZ proteins present in the cultured members of the Sterolibacteriaceae family. Sequence alignments were done with MAFFT 7.407. Phylogenetic trees were calculated based on maximum likelihood (1,000 iterations) with IQ-TREE 1.6.12 (ref. ^79^) with the option –m MFP and visualized using iTOL (6.7.5.)^80^. Alignment of HCO active sites was done using MAFFT 7.407 with a representative of each family from the HCO superfamily and the *norB* sequences retrieved from MAG1 and MAG5.

### Protein extraction and digestion for metaproteomic analyses

On day 1,548, 15 ml of biomass was collected, pelleted by centrifugation at 7,942*g* and 4 °C for 20 min and stored at −80 °C. Biomass pellets were resuspended in 100 µl milliQ water and heated at 95 °C for 5 min with agitation before sonication for 10 min in a sonication bath (Branson 5800 Ultrasonic Cleaner). Samples were centrifuged at 14,000*g* for 20 min at 4 °C, and supernatant was transferred to a new Eppendorf tube as ‘soluble’ extracts and RapiGest SF (Waters) was added to a final concentration of 0.1% (v/v). Pellets were washed with 100 µl milliQ water before addition of 50 µl 2% (v/v) RapiGest SF solution and heated at 95 °C for 5 min. Samples were reduced with bond-breaker TCEP solution (Thermo Fisher Scientific) for 20 min before alkylation with 50 mM chloroacetamide for 20 min at room temperature in the dark. Enzymatic digestion was performed by incubating protein extracts overnight at 37 °C with 0.4 µg LysC (Wako Chemicals Europe) and 0.4 µg trypsin (Promega). RapiGest SF was removed by incubating tryptic digests in 0.5% trifluoroacetic acid at 37 °C for 40 min and subsequent centrifugation at 14,000*g* for 20 min at 4 °C. Samples were analysed both before and after salt-mediated organic solvent precipitation^81^.

### Liquid chromatography with ion mobility spectrometry–tandem mass spectrometry metaproteome analyses and data processing

Tryptic digests were measured in duplicate by nanoflow liquid chromatography (nanoElute; Bruker Daltonics) with online ion mobility spectrometry–tandem mass spectrometry (timsTOF Pro 2; Bruker Daltonics) and real-time protein identification technology using the parallel database search engine in real-time (PaSER 2023; Bruker Daltonics). Peptides were loaded directly onto the C18 reverse-phase analytical column (Bruker FIFTEEN 0.075 mm × 150 mm, 1.9 µm particles, 120 Å pore size, C18 ReproSil AQ; Bruker Daltonics) at a constant pressure of 800 bar and separated using a 60-min-long linear gradient of 3–45% acetonitrile in 0.1% formic acid with 0.02% trifluoroacetic acid at 500 nl min^−1^ at 45 °C. Peptides eluting from the column were analysed by parallel accumulation serial fragmentation in data-dependent acquisition mode (dda-PASEF)^82^ using the default 1.1 second duty cycle method (mass range, 100–1,700 *m*/*z;* mobility range, 0.6–1.6 1/*K*_0_; accumulation time, 100 ms; ramp time, 100 ms; PASEF cycles, 10; dynamic exclusion, 0.4 min). Acquired ion mobility spectrometry–tandem mass spectrometry spectra were streamed directly to the PaSER box for real-time database searching using the ProLuCID (1.3) search engine^83^. Fragmentation spectra were searched against a custom protein sequence database constructed with the protein sequences from MAGs that had a relative abundance above 0.5% in the latest metagenomics sample, corresponding to MAG1 to MAG8, and including 28,576 proteins. The search was constricted by the following settings: 20 ppm precursor mass tolerance, 30 ppm fragment ion mass tolerance, strict tryptic cleavage, maximum of 4 missed cleavages, carbamidomethyl (C) as fixed modification, and deamidation (NQ), oxidation (M) and formylation (K) as variable modifications. Individual search results were combined and validated using DTASelect (2.0) with TIMScore enabled to achieve a ≤1% false discovery rate at the protein level.

### Cell abundance quantification

#### Design and in silico evaluation of 16S rRNA probes

A search on probeBase^84^ revealed no previously available probes that targeted the 16S rRNA gene sequences of *Ca.* Nitricoxidivorans perseverans and *Ca.* Nitricoxidireducens bremensis. Two oligonucleotide probes were designed to target these organisms using the probe design and probe match functions of the ARB software (6.1)^85^ and the SILVA SSU Ref NR 99 138.1 database^77,86^. Full-length (>1,500 bp) 16S rRNA gene sequences extracted from the metagenome were imported into the database and aligned using SINA (SILVA Incremental Aligner 1.2.12)^87^. Probe S-*-Nper-0205-a-A-23 (Nper205, 5′-TGTCGCGCGAGGTCGTTTCCAAT-3′) targeted only *Ca*. Nitricoxidivorans perseverans with no mismatches, and had at least one mismatch with all other sequences in the database and at least five mismatches with the other 16S rRNA sequences extracted from the metagenome. A helper probe (5′-ACTAGCTAATCCGGCATCGGCCGCT-3′) was designed to ensure efficient hybridization efficiency of Nper205 to the target organisms. Probe S-*-Nbre-0448-a-A-19 (Nbre448, 5′-TTAGCGACGACCGTTTCGT-3′) targeted with no mismatches *Ca*. Nitricoxidireducens bremensis and five other uncultured organisms in the database that were not present in our enrichment culture, and had at least one mismatch with all other sequences in the database and at least three mismatches with the other 16S rRNA sequences extracted from the metagenome. The optimal formamide concentrations for the hybridization of the Nper205 and Nbre448 probes were determined from probe dissociation profiles^88^ generated with the image analysis software daime^89^.

#### Cell fixation

Biomass freshly collected from the bioreactor on day 1,256, and sludge collected on the same day and location as the inoculum for the enrichment culture, was fixed with 2% paraformaldehyde as described elsewhere^90^. Fixed samples were filtered onto 0.22 μm polycarbonate filters (Millipore) and stored at −20 °C until analysis.

#### Visualization and quantification of cells

To estimate the abundance of *Ca*. Nitricoxidivorans perseverans and *Ca*. Nitricoxidireducens bremensis in the enrichment culture and the denitrifying sludge from which it was inoculated, CARD–FISH was performed on filter pieces with cells from the enrichment culture and the inoculum using Nper205 and Nbre448 probes as described elsewhere^91^. Nper205 and Nbre448 probes required formamide concentrations in the hybridization buffer of 40% and 30% (v/v), respectively. Positive and negative controls with an equimolar mixture of probes EUB338-I, EUB338-II and EUB338-III^92,93^, and with probe NON338 (ref. ^94^) were included. Following CARD–FISH, cells were stained using DAPI to target DNA of all microorganisms. Relative abundances of *Ca*. Nitricoxidivorans perseverans and *Ca*. Nitricoxidireducens bremensis were determined from CARD–FISH and DAPI counts (*n* ≥ 1,000) in triplicate filter pieces that were either hybridized with Nper205 or with Nbre488. Double CARD–FISH was performed to simultaneously visualize cells of *Ca*. Nitricoxidivorans perseverans and *Ca*. Nitricoxidireducens bremensis by hybridization with probe Nper205 followed by inactivation of peroxidases and a second hybridization step with probe Nbre448. The Zeiss Axio Imager M2 epifluorescence microscope equipped with an Axiocam 506 mono camera (Zeiss) was used for cell counting and image acquisition.

### Etymology

*Ca.* Nitricoxidivorans (ni.tric.o.xi.di.vo’rans. N.L. neut. n. *nitricum oxidum*, NO; L. pres. part. *vorans*, eating; N.L. part. adj. *nitricoxidivorans*, eating NO, based on its feeding on NO). The type species of the genus is *Ca.* Nitricoxidivorans perseverans.

*Ca*. Nitricoxidivorans perseverans (per.se’ve.rans. L. part. adj. *perseverans*, perseverant, referring to its constant presence in the enrichment culture).

*Ca.* Nitricoxidireducens (ni.tric.o.xi.di.re.du’cens. N.L. neut. n. *nitricum oxidum*, NO; L. v. reducere, reduce; N.L. masc. part. n. Nitricoxidireducens, reducing NO). The type species of the genus is *Ca.* Nitricoxidireducens bremensis.

*Ca*. Nitricoxidireducens bremensis (bre.men’sis. M.L. masc. adj. bremensis, belonging to the German city Bremen, due to its enrichment from a location in Bremen, Germany).

### Reporting summary

Further information on research design is available in the Nature Portfolio Reporting Summary linked to this article.

### Supplementary information

Supplementary Information Supplementary Information, Figs. 1–7, Tables 1–7 and References.

Reporting Summary

### Source data

Source Data Fig. 1 Rates of influent and effluent NO, formate and N_2_O in the bioreactor.

## Data Availability

Raw data from metagenomic and metatranscriptomic analyses and all MAGs generated in this study have been deposited in the NCBI under BioProject number PRJNA849246. The MAGs of *Ca*. Nitricoxidivorans perseverans (MAG1) and *Ca*. Nitricoxidireducens bremensis (MAG5) are deposited, respectively, under BioSample numbers SAMN30388482 and SAMN30388483 and genome accession numbers CP107246 and JAOTRT000000000. Metaproteomics data, including raw data files and ProLuCID search results, have been deposited to the ProteomeXchange Consortium via the PRIDE database^95^ under identifier PXD037586. Databases used in this study are SILVA SSU Ref NR 99 138.1, NR (GenBank 249; NCBI), Pfam 35.0, UniProt release 2022_01, KEGG release 101.0, GTDB release 207, GEM 2021 and FunGene 7.3. Source data are provided with this paper.
