# Publisher Correction: Enrichment and characterization of a nitric oxide-reducing microbial community in a continuous bioreactor

**DOI:** 10.1038/s41564-024-01602-3

**Published:** 2024-02-02

**Authors:** Paloma Garrido-Amador, Niek Stortenbeker, Hans J. C. T. Wessels, Daan R. Speth, Inmaculada Garcia-Heredia, Boran Kartal

**Affiliations:** 1 Max Planck Institute for Marine Microbiology, Bremen, Germany; 2 Translational Metabolic Laboratory, Department of Laboratory Medicine, Radboud University Medical Center, Nijmegen, The Netherlands; 3 School of Science, Constructor University, Bremen, Germany

**Keywords:** Microbiology, Bacterial physiology

Correction to: *Nature Microbiology* 10.1038/s41564-023-01425-8, published online 10 July 2023.

In the version of this article initially published, there were errors in the second column of Table 3: in the first, third and fifth rows under the “N_2_O reduction” heading, “N_2_O” appeared as “NO”. The errors have been corrected in the HTML and PDF versions of the article.

