# Supplementary material for: Enrichment and characterization of a nitric oxide-reducing microbial community in a continuous bioreactor
Source: Nat Microbiol. 2023 Jul 10;8(8):1574–86. doi: 10.1038/s41564-023-01425-8 (PMC10390337; doi:10.1038/s41564-023-01425-8)
Supplement: Supplementary file 1 — Supplementary Information, Figs. 1–7, Tables 1–7 and References. [file 41564_2023_1425_MOESM1_ESM.pdf]

nature microbiology

Article

https://doi.org/10.1038/s41564-023-01425-8

Enrichment and characterization of a nitric oxide-reducing microbial community in a continuous bioreactor

In the format provided by the authors and unedited

SUPPLEMENTARY INFORMATION

Taxonomic classification of MAG1 and MAG5

The taxonomy of MAG1 and MAG5 was investigated combining whole-genome classification tools with 16S rRNA gene sequence identity, average amino acid identity (AAI), and average nucleotide identity (ANI) analyses.

With a 16S rRNA gene sequence identity of 96.53%, MAG1 had *Georgfuchsia toluolica* as its closest representative. In the case of MAG5, its closest representative was *Sulfuritalea hydrogenivorans* with 96.55% 16S rRNA gene sequence identity. Both of these microorganisms belong to the *Sterolibacteriaceae* family, which was formerly a part of the *Rhodocyclaceae* family. The *Rhodocyclaceae* was recently re-defined and several of its genera, including *Georgfuchsia* and *Sulfuritalea*, were transferred from the *Rhodocyclales* to the *Nitrosomonadales* order and grouped into a new family named *Sterolibacteriaceae*[1], accepted by the International Committee on Systematics of Prokaryotes (ICSP) and adopted by the NCBI. Average nucleotide identity (ANI) and average amino acid identity (AAI) analyses (Supplementary Tables S3 and S4) suggested differently than the 16S rRNA gene and indicated that both MAG1 and MAG5 were more closely related to a different organism from the *Sterolibacteriaceae* family, *Sulfurisoma sediminicola*. AAI values of MAG1 and MAG5 with *Sulfurisoma sediminicola*, *Sulturitalea hydrogenovorans* and *Denitratisoma oestradiolicum* were higher than the genus criterion level[2,3] and complicated their taxonomic classification, as it became unclear which genus these organisms would belong to. Meanwhile, the GTDB-Tk classified MAG1 and MAG5 as a new genus within the *Rhodocyclaceae* family (i.e. given that the GDTB taxonomy does not include the *Sterolibacteriaceae* family) and new species of the *Sulfurisoma* genus, respectively. The classification of MAG5 as a species within *Sulfurisoma* is, however, problematic, as phylogenetic trees based on 16S rRNA sequences of the *Sterolibacteriaceae* family with MAG1 and MAG5 (Supplementary Fig. S3) place MAG5 in a branch with *Sulfuritalea* and separate from *Sulfurisoma*, resulting in a loss of monophyleticity of the *Sulfurisoma* genus. Taken together, MAG5 should not be placed into an existing genus but in a new one, and thus we propose MAG1 and MAG5 as novel species of novel genera within the *Sterolibacteriaceae* family with the names of *Candidatus* Nitricoxidivorans perseverans and *Candidatus* Nitricoxidireducens bremensis, respectively.

Potential chemolitoautotrophy of *Ca.* Nitricoxidivorans perseverans and *Ca.* Nitricoxidireducens bremensis

In addition to denitrification and the respiration of formate, *Ca*. N. perseverans and *Ca.* N. bremensis had the genomic potential for chemolithotrophy through the oxidation of hydrogen and reduced sulfur compounds (Supplementary Tables S5 and S6). Both organisms encoded and expressed genes to synthesize at least four different types of hydrogenases and multiple proteins related to sulfur metabolism, suggesting the capacity of these organisms to transform different sulfur compounds. A detailed analysis of these pathways is beyond the scope of this study, but the presence and expression of these genes in *Ca*. N. perseverans and *Ca.* N. bremensis suggest that they might have the versatile metabolisms to respire small organic compounds, hydrogen and sulfur compounds.

SUPPLEMENTARY FIGURES

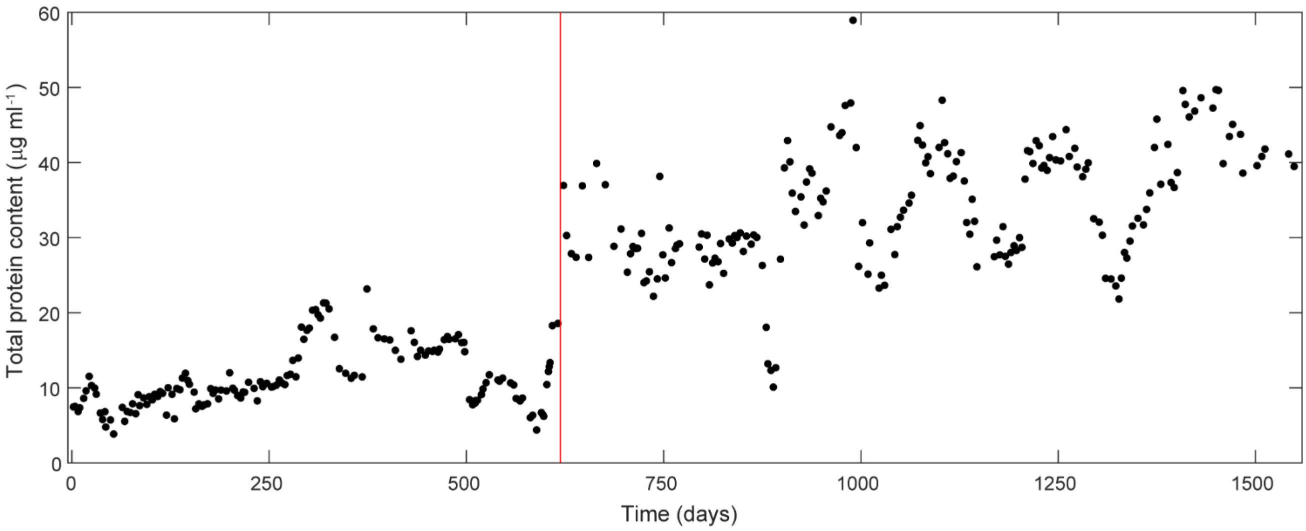

Figure S1. Protein concentrations in the NO-reducing enrichment culture. Until day 616 (indicated by a red line), a protein quantification method based on the Bradford assay was used. From that day onwards, proteins were measured following the BCA method. Decreases observed in the protein concentrations were due to excessive biomass sampling. When the sampling periods ended and the culture was not disturbed, protein levels in the enrichment culture recovered.

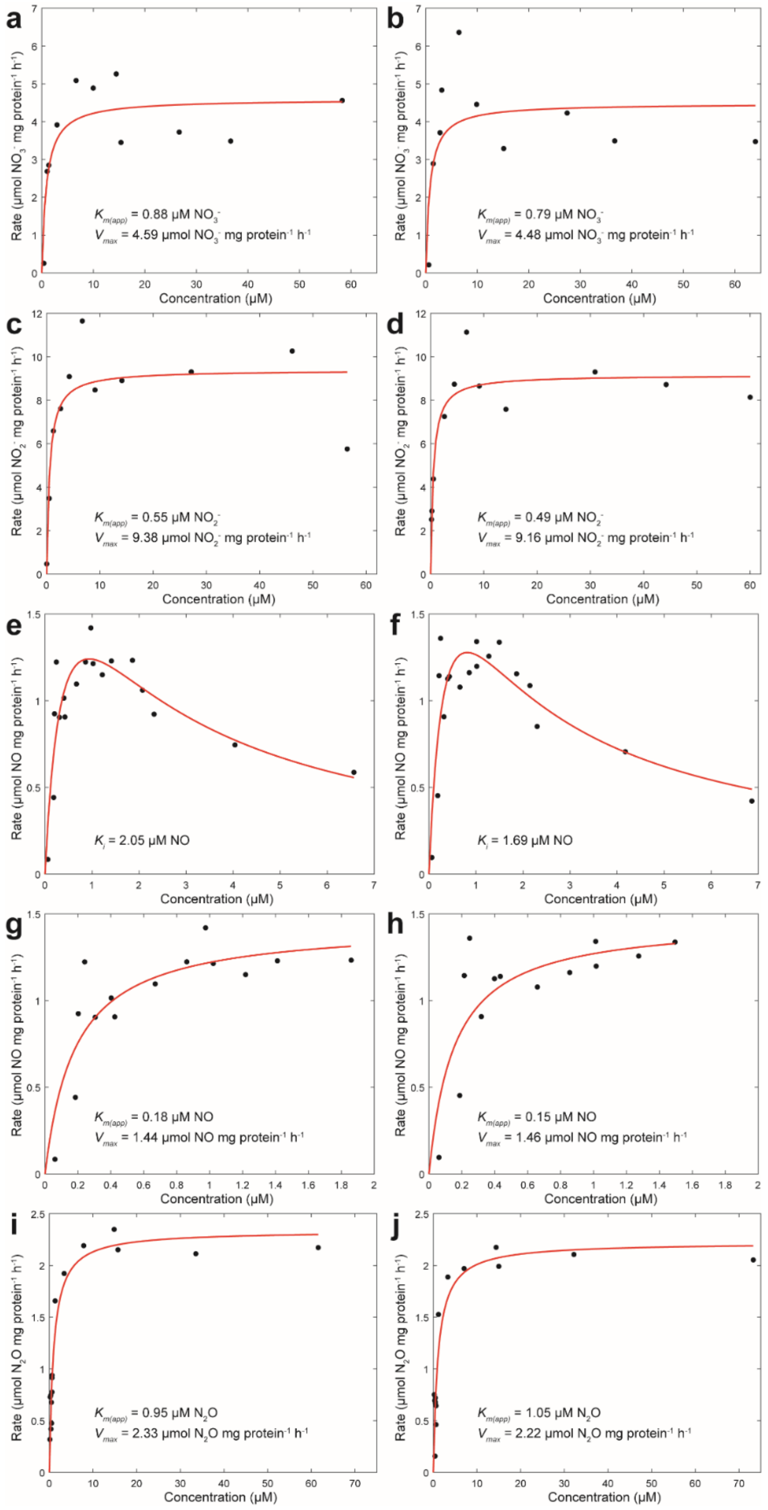

Figure S2. Substrate reduction kinetics of the NO-reducing enrichment culture. Graphics on the left and right of the figure represent the results obtained from duplicate experiments. Closed circles indicate N-oxide reduction rates observed experimentally, while red lines indicate the fitted Monod equations (in a, b, c, d, g, h, I, j) or the fitted Haldane equation (e, f). V_{max} (µmol N-oxide mg protein^{-1} h^{-1}) indicates the maximum reduction rate, $K_{m(app)}$ (µM) indicates the apparent half saturation constant (both calculated with the Michaelis-Menten equation), and K_i (µM) indicates the substrate inhibition constant (calculated with the Haldane equation).

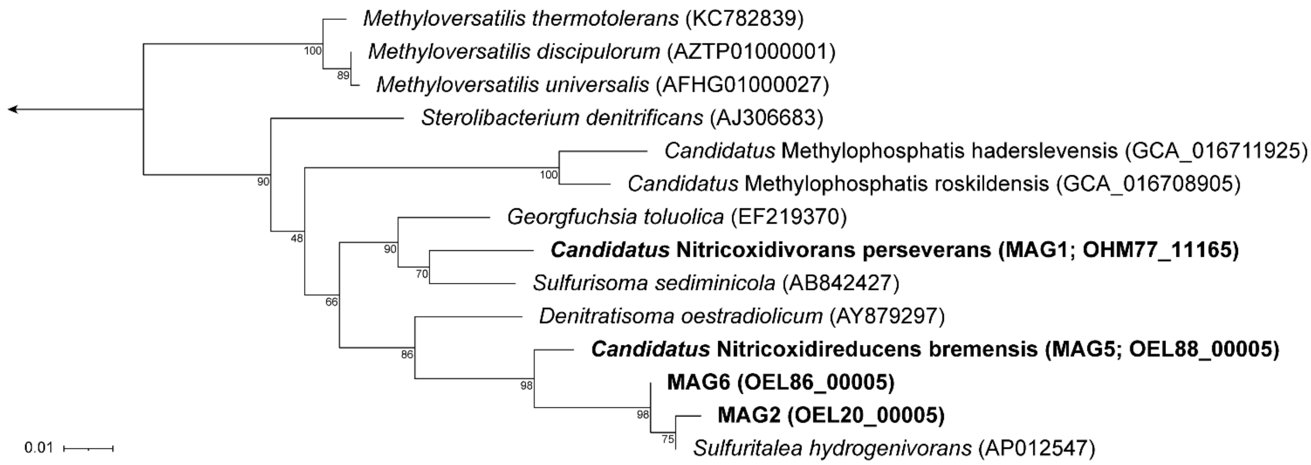

Figure S3. Phylogenetic affiliation of *Candidatus* Nitricoxidivorans perseverans (MAG1) and *Candidatus* Nitricoxidireducens bremensis (MAG5) based on 16S rRNA gene sequences of cultured members of the *Sterolibacteriaceae* family. The organisms obtained in this study are indicated in bold. Accession numbers of the 16S rRNA gene sequences of *Ca*. Methylophosphatis haderslevensis and *Ca*. Methylophosphatis roskildensis were not available and instead, accession numbers of their genome assembly from which the 16S rRNA gene sequence was extracted is provided. The genus *Thauera* (MH251633, KX953213, AB681853, AB681922, KY425607, AJ315677, AF123264, X77118) was used as outgroup. The tree was calculated based on maximum likelihood (1,000 iterations) using IQtree. Ultrafast bootstrap values[4] are indicated at the branch nodes. Scale bar indicates 0.01 estimated substitutions per nucleotide.

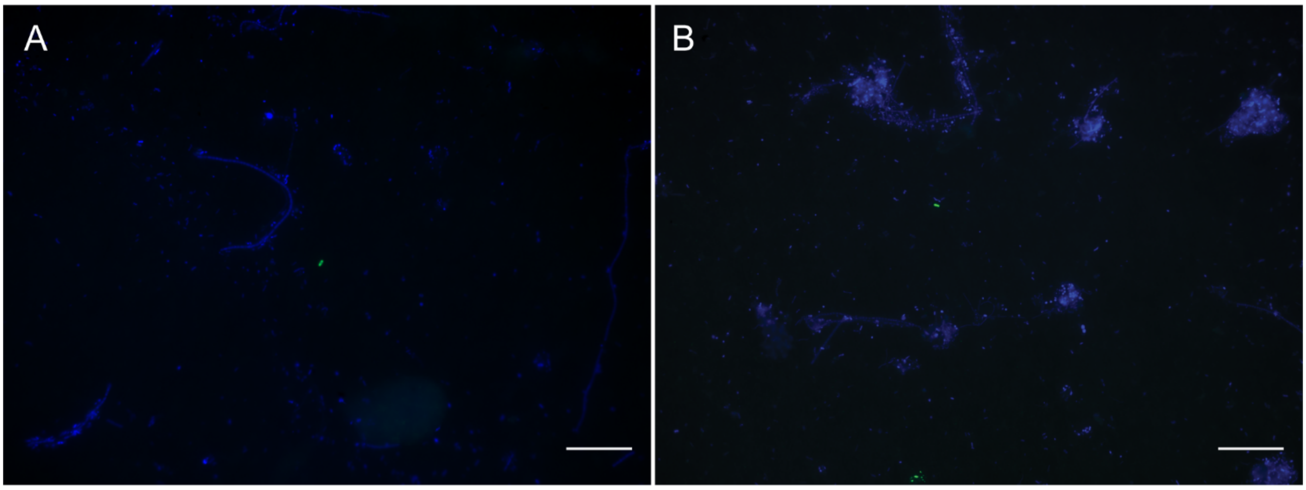

Figure S4. Visualization of *Candidatus* Nitricoxidivorans perseverans and *Candidatus* Nitricoxidireducens bremensis in sludge collected from the municipal wastewater treatment plant in Bremen, Germany. A) Cells hybridized with probe Nper205 (green) corresponded to *Ca.* Nitricoxidivorans perseverans while B) cells that hybridized with probe Nbre448 (green) corresponded to *Ca.* Nitricoxidireducens bremensis. All cells were stained with DAPI (blue). Experiments were performed in triplicate using 3 filtered wastewater treatment plant sludge samples. Scale bar: 20 µm.

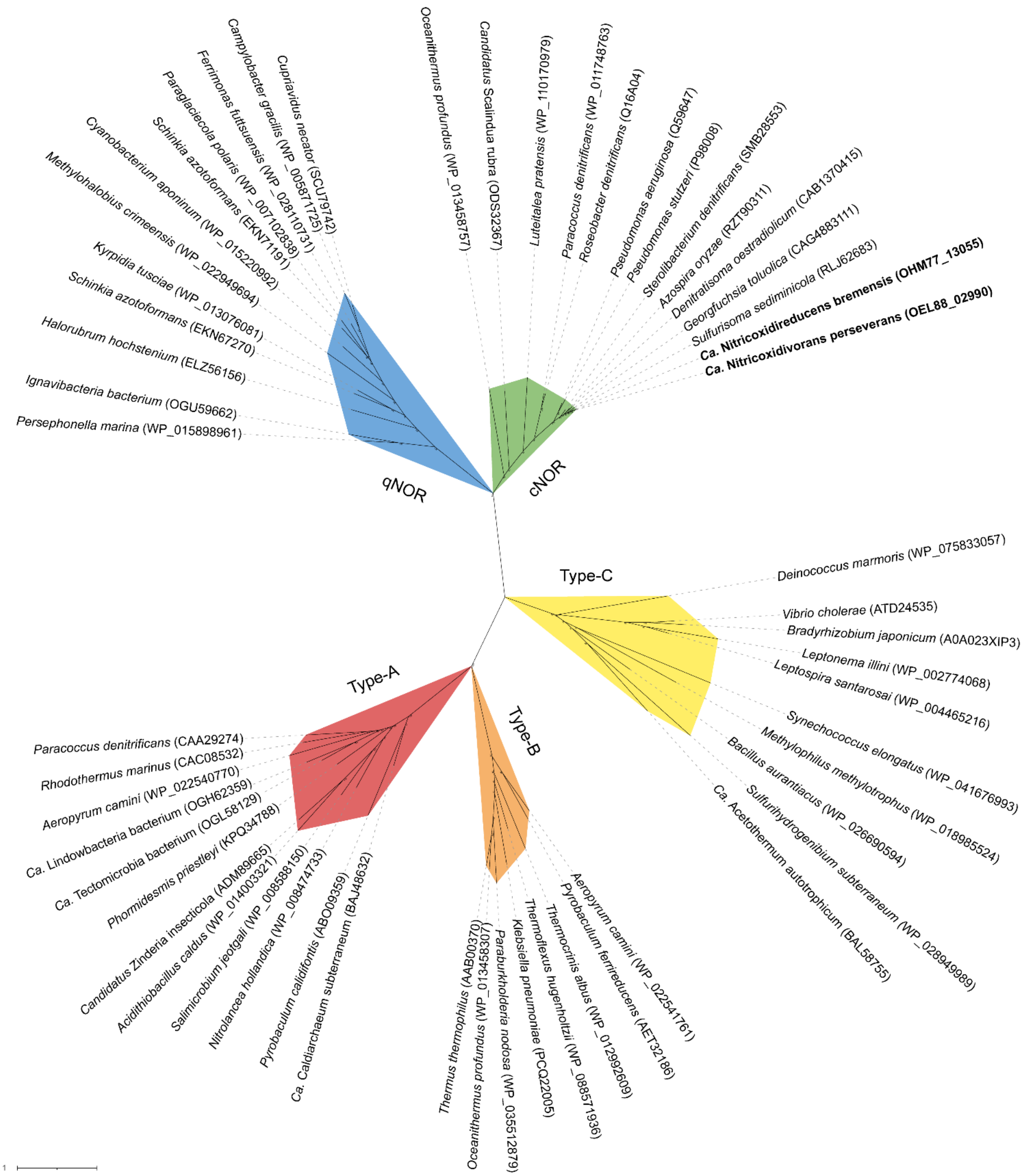

Figure S5. Phylogeny of heme-copper oxidases (HCOs) families. Families of oxygen reductases are presented in red (A-type family; cytochromes *aa3* and *caa3*), orange (B-type family; cytochrome *ba3*) and yellow (C-type family; cytochrome *cbb3*), and nitric oxide reductases are presented in blue (qNOR), and dark green (cNOR). NOR sequences from *Candidatus* Nitricoxidivorans perseverans and *Candidatus* Nitricoxidireducens bremensis are indicated in bold. The tree was calculated using 530 amino acid positions. The tree was calculated based on maximum likelihood (1,000 iterations) using IQtree. Ultrafast bootstrap values[4] are indicated at the branch nodes. Scale bar indicates 1 estimated substitutions per site.

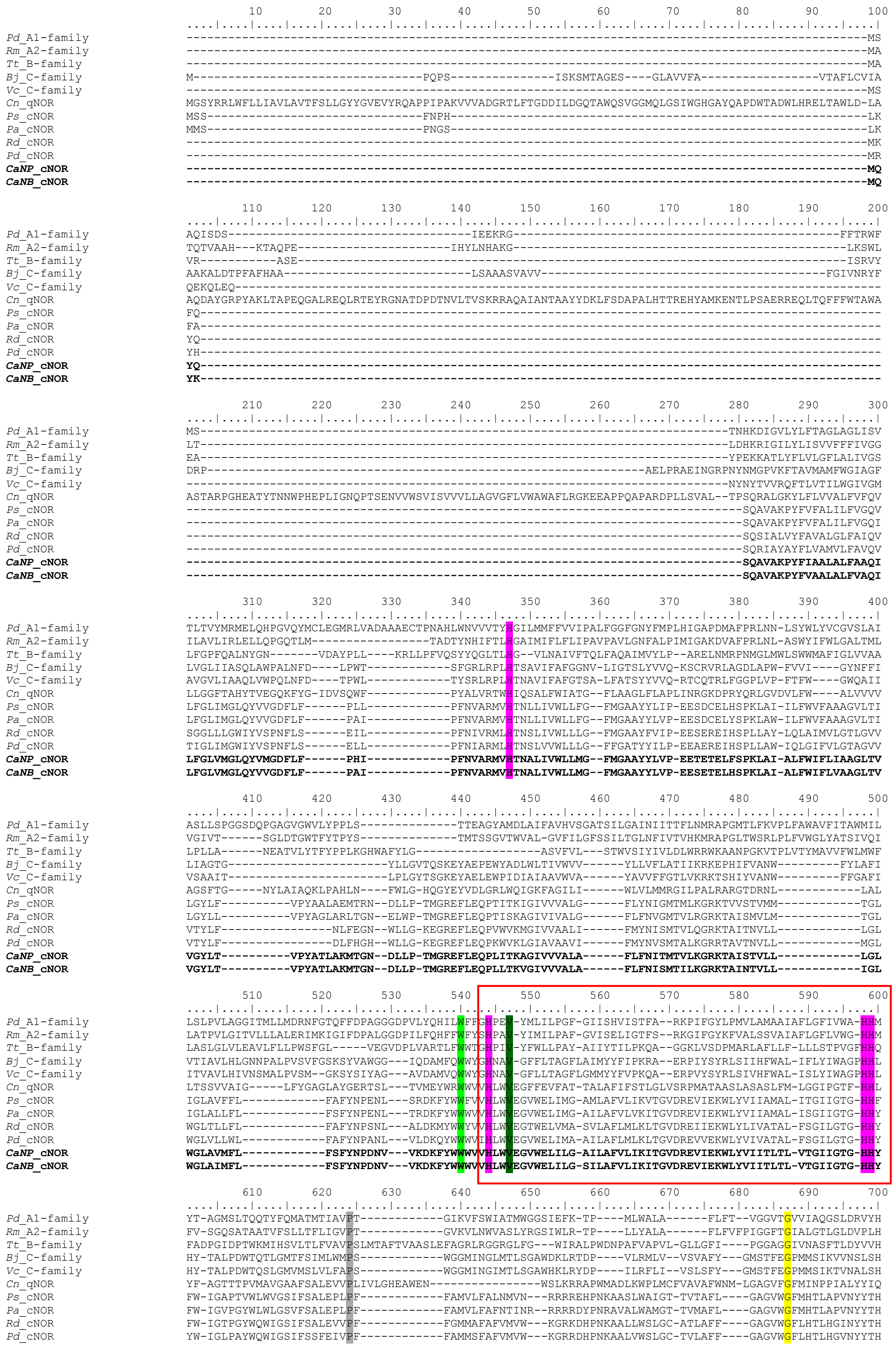

Pd_A1-family
Rm_A2-family
Tt_B-family
Bj_C-family
Vc_C-family
Cn_qNOR
Ps_cNOR
Pa_cNOR
Rd_cNOR
Pd_cNOR
CaNP_cNOR
CaNB_cNOR

```
CaNP_cNOR          YW-IGTPEYWQVWGSIFSALEPIPF------------FAMTLFAFNMVN---RRRREHPNKAATLWALGT-GVMSFL----GAGVWGFLHTLAPVNFYTH
CaNB_cNOR          YW-IGTPEYWQIWGSIFSALEPIPF------------FAMTLFAFNMVN---RRRREHPNKAATLWALGT-GVMSFL----GAGVWGFLHTLAPVNFYTH

                            710       720       730       740       750       760       770       780       790       800
                   ....|....|....|....|....|....|....|....|....|....|....|....|....|....|....|....|....|....|....|....|
Pd_A1-family       DTYYIVAHFHYVMSLGALFAIFAGTYYWIGKMSG-----RQYPEWAGQLHFWMMFIGSNLIF-FPQHFLGRQG----------MPRR----------YI-
Rm_A2-family       DTYFVVAHFHYVMVSGGLLAFLGGLHYWWPKMFG-----RLYNEKLAQIAALLIFVGFNVTF-FPQFILGTQG----------MPRR----------YF-
Tt_B-family        NTAWVPGHFHLQVASLVTLTAMGSLYWLLPNLTGK--PISDAQRRLGLAVVWLWFLGMMIMA-VGLHWAGLLN----------VPRR---------AYIA
Bj_C-family        YTDWTIGHVHSGALGWVGFVSFGALYCLVPWVWN---RKGLYSLKLVNWHFWIATLGIVLYI-SAMWVSGILQ--------GLMWRAYTSLGFLEYSFI-
Vc_C-family        YTDWTIGHVHSGALGWVAMVSIGSVYHLIPRLFG---QERMYSVSLINAHFWLATIGTVLYI-VAMWISGVMQ--------GLMWRAVNSDGTLTYSFV-
Cn_qNOR            GQNTTPVHAHAALFGVYGFLALGFTLLVLRYVRP----AYRLSPALMKTAFWGLNLGLVLMIGTSLLPIGIIQFLASVEHGTWYARSE--------AFM-
Ps_cNOR            GSQLTAAHGHLAFYGAYAMIVMTMISYAMPRLRGLGEAPDARAQRIEVWGFWLMTISMIAIT-LFLTAAGVVQ--------IWLQRIPAD--GAAMSFM-
Pa_cNOR            GTQLTAAHGHMAFYGAYAMIVMTIISYAMPRLRGIGEAMDNRSQVLEMWGFWLMTVAMVFIT-LFLSAAGVLQ--------VWLQRMPAD--GAAMTFM-
Rd_cNOR            GTQITAAHGHLAFFGAYVSLNLAIFSYAFPILRK----RDPYNQVLNMASFWLMAGGMTFMT-FVLTFAGTVQ--------THAQRVQGD------YFM-
Pd_cNOR            GTQITAAHGHLAFYGAYVCLVLALVTYCMPLMKN----RDPYNQVLNMASFWLMSSGMVFMT-VTLTFAGTVQ--------THLQRVEGG------FFM-
CaNP_cNOR          GTQITAAHGHMAFYGAYVMVVLTIISYAMPLMRGRA-ANSNKSQVVEMWGFWLMTVSIVFIT-LFLTAAGILQ--------VWLQRVSDN----PMPFM-
CaNB_cNOR          GTQITAAHGHMAFYGAYVMVVLTIISYAMPLMRGRA-ANSNKAQVVEMWGFWLMTVSIVFIT-LFLTGAGILQ--------VWLQRISDN----PMPFM-

                            810       820       830       840       850       860       870       880       890       900
                   ....|....|....|....|....|....|....|....|....|....|....|....|....|....|....|....|....|....|....|....|
Pd_A1-family       ---DYPVEFSYWNNISSIGAYISFASFLFFIGIVFYTLFAGKPVNVPNYWNEHADTLEWT----LPSPPPEHTFETLP----KPEDWDRA----------
Rm_A2-family       ---DYVPEFTTLHQLSTVGSWILGLGLLLVAVCLLHSLLKGQQAPA-NPWG--AGTLEWTHTGRLPSP---HNFERTPVVTRGPYDYHLAEEIFGNGHGD
Tt_B-family        QVPDAYPHAAVPMVFNVLAGIVLLVALLLFIYGLFSVLLSRERKPE-------------LAEAPLPFA------EVIS----GPEDRRLVLAMDRIGFWF
Bj_C-family        ---ESVEAMHPFYIIRAAGGGLFLIGALIMAYNLWMTVRVGEQEVQ-------------MPVALQPAE--------------------------------
Vc_C-family        ---ESVQASYPFYTVRFIGGFIFLSGMFLMAYNAYKTISAPKNSLK-------------AIV--QPA---------------------------------
Cn_qNOR            ---Q-QPILQTLRWVRTFGDVVFIVGAVSFAWQVVLGLANRTPLAAD------------AATARMKTA--------------------------------
Ps_cNOR            ---NTADQLAIFFWLRFIAGVFFLIGLVCYLYSFRQ---RGRVPVV------------------VAAP--------------------------------
Pa_cNOR            ---ATQDQLAIFYWLREGAGVVFLIGLVAYLLSFR----RGKAAA-------------------------------------------------------
Rd_cNOR            ---DVQDAITIFYWMRFGSGIAVVLGALLFIYAVAV---PRKEIIS-------------------PSDKQP-----------------------------
Pd_cNOR            ---DVQDGLALFYWMRFGSGVAVVLGALLFIYAVLF---PRREVVK-------------------AGPVQAHK---------------------------
CaNP_cNOR          ---VAQDQVSLFYWMREWTGVVFLVGLVVYLWSFFV---KGEEKSA------------------------------------------------------
CaNB_cNOR          ---VAQEQANLFYWMREWTGVAFLVGLVVYLASFFI---KGEEKAT------------------------------------------------------

                            910       920       930
                   ....|....|....|....|....|....|.
Pd_A1-family       ---------------------------QAHR
Rm_A2-family       GSSTPAVI---------------PAQGQAQS
Tt_B-family        AVAAILVVLAYGPTLVQLFGHLNPVPGWRLW
Bj_C-family        -------------------------------
Vc_C-family        -------------------------------
Cn_qNOR            -----------------------------VR
Ps_cNOR            ----------------------------AAA
Pa_cNOR            -------------------------------
Rd_cNOR            ---------------------------VAAE
Pd_cNOR            ------------------DGHL-----EAAE
CaNP_cNOR          -------------------------------
CaNB_cNOR          ------------------------------A
```

Figure S6. Amino acid sequence analysis of norB from *Candidatus* Nitricoxidivorans perseverans and *Candidatus* Nitricoxidireducens bremensis. Included are sequences of oxygen reductases (A-type family: cytochromes aa3 and caa3, B-type family: cytochrome ba3, and C-type family: cytochrome cbb3) and nitric oxide reductases (qNOR and cNOR) of the heme-copper oxidase (HCO) superfamily from *Paracoccus denitrificans* (*Pd*_A1-family; CAA29274), *Rhodothermus marinus* (*Rm*_A2-family; CAC08532), *Thermus thermophilus* (*Tt*_B-family; AAB00370), *Bradyrhizobium japonicum* (*Bj*_C-family; A0A023XIP3), *Vibrio cholera* (*Vc*_C-family; ATD24535), *Cupriavidus necator* (*Cn*_qNOR; SCU79742), *Pseudomonas stutzeri* (*Ps*_cNOR; P98008), *Pseudomonas aeruginosa* (*Pa*_cNOR; Q59647), *Roseobacter denitrificans* (*Rd*_cNOR; Q16A04), *Paracoccus denitrificans* (*Pd*_cNOR; WP_011748763), and the organisms obtained in this study (in bold), *Candidatus* Nitricoxidivorans perseverans (*CaNP*_cNOR; OHM77_13055) and *Candidatus* Nitricoxidireducens bremensis (*CaNB*_cNOR; OEL88_02990). Amino acid residues that are conserved in all families of HCO are highlighted in different colors. Transmembrane helices VI and VII, which form the active site of proteins within HCO families, are indicated with a red square.

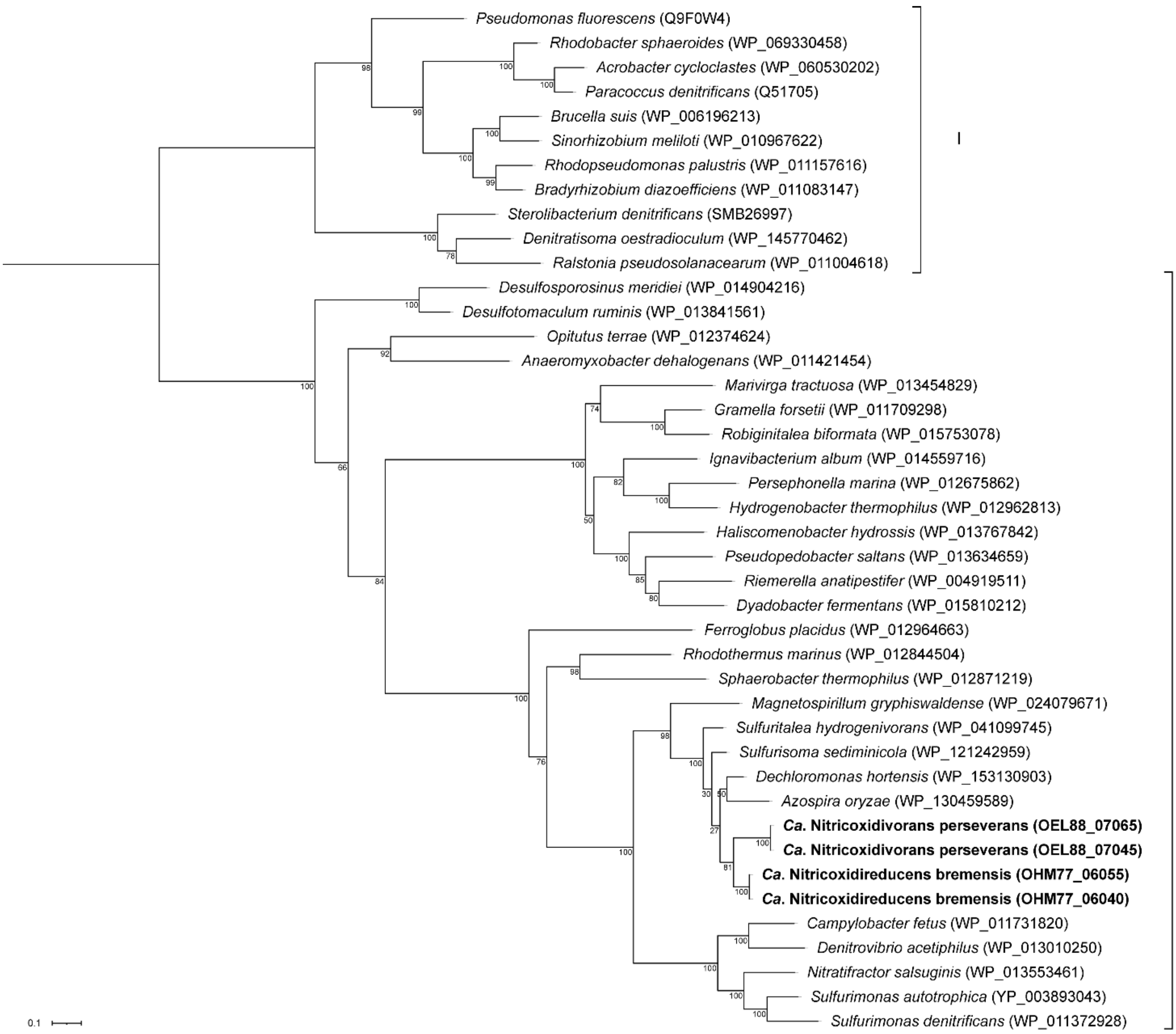

Figure S7. Phylogeny of NosZ. Sequences belonging to clade I and clade II are indicated on the right. NosZ sequences from *Candidatus* Nitricoxidivorans perseverans and *Candidatus* Nitricoxidireducens bremensis are indicated in bold. The tree was calculated with IQtree using 750 amino acid positions, based on maximum likelihood (1,000 iterations). Ultrafast bootstrap values[4] are indicated at the branch nodes. Scale bar indicates 0.1 estimated substitutions per site.

SUPPLEMENTAL REFERENCES:

1. Boden, R., Hutt, L. P. & Rae, A. W. 2017. Reclassification of Thiobacillus aquaesulis (Wood & Kelly, 1995) as Annwoodia aquaesulis gen. nov., comb. nov., transfer of Thiobacillus (Beijerinck, 1904) from the Hydrogenophilales to the Nitrosomonadales, proposal of Hydrogenophilalia class. nov. within the 'Proteobacteria', and four new families within the orders Nitrosomonadales and Rhodocyclales. *International Journal of Systematic and Evolutionary Microbiology* **67**, 1191–1205 (2017).
2. Konstantinidis, K. T. & Tiedje, J. M. Prokaryotic taxonomy and phylogeny in the genomic era: advancements and challenges ahead. *Current Opinion in Microbiology* **10**, 504–509 (2007).
3. Konstantinidis, K. T., Rosselló-Móra, R. & Amann, R. Uncultivated microbes in need of their own taxonomy. *ISME J* **11**, 2399–2406 (2017).
4. Hoang, D. T., Chernomor, O., von Haeseler, A., Minh, B. Q. & Vinh, L. S. UFBoot2: improving the ultrafast bootstrap approximation. *Molecular Biology and Evolution* **35**, 518–522 (2018).

Acc. number	Sample type	Sampling day	Sequencing platform	Sequencing depth (Gb)	Raw read number	Average sequence length (bp)	Estimated coverage	Total assembly length (Mbp)	Number of contigs	Max. contig length (Mbp)
SRR20819805	Metagenome	497	Illumina HiSeq3000	5	43080991	146.5	20x	-	-	-
SRR20819804	Metagenome	497	PacBio Sequel	7	645735	10823	10x	29.16	861	2.7
SRR20819803	Metagenome	1269	Illumina HiSeq3000	20	143893996	142.8	200x	115.46	130815	1.7
SRR20819802	Metatranscriptome	1304	Illumina HiSeq3000	40	293734864	142.5	400x	-	-	-

Table S1. Characteristics of metagenome and metatranscriptome samples obtained from the enrichment culture and included in this study.

MAG ID	NCBI taxonomy	GDTB taxonomy
MAG1	Bacteria; Proteobacteria; Betaproteobacteria; Nitrosomonadales; Sterolibacteriaceae	d__Bacteria;p__Proteobacteria;c__Gammaproteobacteria;o__Burkholderiales;f__Rhodocyclaceae;g__;s__
MAG5	Bacteria; Proteobacteria; Betaproteobacteria; Nitrosomonadales; Sterolibacteriaceae	d__Bacteria;p__Proteobacteria;c__Gammaproteobacteria;o__Burkholderiales;f__Rhodocyclaceae;g__Sulfurisoma;s__
MAG6	Bacteria; Proteobacteria; Betaproteobacteria; Nitrosomonadales; Sterolibacteriaceae; Sulfuritalea	d__Bacteria;p__Proteobacteria;c__Gammaproteobacteria;o__Burkholderiales;f__Rhodocyclaceae;g__Sulfuritalea;s__Sulfuritalea hydrogenivorans
MAG2	Bacteria; Proteobacteria; Betaproteobacteria; Nitrosomonadales; Sterolibacteriaceae; Sulfuritalea	d__Bacteria;p__Proteobacteria;c__Gammaproteobacteria;o__Burkholderiales;f__Rhodocyclaceae;g__Sulfuritalea;s__
MAG7	Bacteria; Proteobacteria; Betaproteobacteria; Rhodocyclales; Rhodocyclaceae; Azospira	d__Bacteria;p__Proteobacteria;c__Gammaproteobacteria;o__Burkholderiales;f__Rhodocyclaceae;g__Azospira;s__Azospira suillum
MAG3	Bacteria; Proteobacteria; Deltaproteobacteria; Desulfobacterales; Desulfocapsaceae; Desulforhopalus	d__Bacteria;p__Desulfobacterota;c__Desulfobulbia;o__Desulfobulbales;f__Desulfocapsaceae;g__Desulforhopalus;s__
MAG8	Bacteria; Proteobacteria; Alphaproteobacteria; Rhodospirillales	d__Bacteria;p__Proteobacteria;c__Alphaproteobacteria;o__Rhodospirillales;f__WMHbin7;g__WMHbin7;s__
MAG11	Bacteria; Proteobacteria; Deltaproteobacteria; Desulfuromonadales; Geobacteraceae; Trichlorobacter	d__Bacteria;p__Desulfobacterota_F;c__Desulfuromonadia;o__Geobacterales;f__Pseudopelobacteraceae;g__Trichlorobacter;s__
MAG10	Bacteria; Proteobacteria; Epsilonproteobacteria; Campylobacterales; Thiovulaceae; Sulfurimonas	d__Bacteria;p__Campylobacterota;c__Campylobacteria;o__Campylobacterales;f__Sulfurimonadaceae;g__Sulfurimonas;s__
MAG4	Bacteria; Firmicutes; Clostridia; Eubacteriales; Eubacteriales; Family XII. Incertae Sedis; Acidaminobact e	d__Bacteria;p__Firmicutes_A;c__Clostridia;o__Peptostreptococcales;f__Acidaminobacteraceae;g__Acidaminobacter;s__Acidaminobacter sp009881125
MAG12	Bacteria	d__Bacteria;p__AABM5-125-24;c__BMS3BBIN04;o__;f__;g__;s__
MAG9	Bacteria; Proteobacteria; Alphaproteobacteria; Hyphomicrobiales; Ancalomicrobiaceae; Siculibacillus	d__Bacteria;p__Proteobacteria;c__Alphaproteobacteria;o__Rhizobiales;f__Ancalomicrobiaceae;g__Siculibacillus;s__

Table S2. Equivalence between taxonomic classification of NCBI and GTDB of MAGs produced in this study.

	MD	MT	MU	SD	SS	SH	DO	GT	**NP**
MT	83.29								
MU	87.22	83.05							
SD	76.12	75.56	76.15						
SS	76.83	75.82	76.67	77.26					
SH	76.16	75.58	76.13	77.10	79.70				
DO	75.21	74.96	75.44	76.24	76.88	76.74			
GT	75.43	75.17	75.42	76.22	76.97	76.86	76.18		
**NP**	**76.33**	**75.53**	**76.19**	**76.95**	**79.95**	**78.26**	**76.77**	**76.49**	
**NB**	**76.32**	**75.51**	**76.23**	**76.90**	**80.18**	**78.51**	**77.70**	**76.39**	**79.99**

Table S3. Average nucleotide identity (ANI) of organisms within the *Sterolibacteriaceae* family and the organisms described in this study (in bold). MD: Methyloversatilis discipulorum; MT: Methyloversatilis thermotolerans; MU: Methyloversatilis universalis; SD: Sterolibacterium denitrificans; SS: Sulfurisoma sediminicola; SH: Sulturitalea hydrogenovorans; DO: Denitratisoma oestradiolicum; GT: Georgfuchsia toluolica; NP: Ca. Nitricoxidivorans perseverans; NB: Ca. Nitricoxidireducens bremensis.

	MD	MT	MU	SD	SS	SH	DO	GT	**NP**
MT	80.46								
MU	85.41	79.64							
SD	57.04	57.08	57.41						
SS	52.04	51.4	52.26	54.39					
SH	57.18	57.34	57.8	62.63	68.45				
DO	57.08	57.15	57.39	63.21	65.8	64.81			
GT	56.04	55.81	56.2	60.32	63.49	62.35	64.52		
**NP**	**58.23**	**58.19**	**58.49**	**63.25**	**70**	**66.77**	**65.29**	**62.99**	
**NB**	**58.27**	**58.56**	**58.86**	**63.55**	**70.9**	**68.08**	**66.06**	**63.09**	**70.68**

Table S4. Average aminoacid identity (AAI) of organisms within the *Sterolibacteriaceae* family and the organisms described in this study (in bold). MD: Methyloversatilis discipulorum; MT: Methyloversatilis thermotolerans; MU: Methyloversatilis universalis; SD: Sterolibacterium denitrificans; SS: Sulfurisoma sediminicola; SH: Sulturitalea hydrogenovorans; DO: Denitratisoma oestradiolicum; GT: Georgfuchsia toluolica; NP: Ca. Nitricoxidivorans perseverans; NB: Ca. Nitricoxidireducens bremensis.

Locus_tag	Length (bp)	Gene name	Product (annotation)	Abudance in transcriptome (RPKM)	Abudance in proteome (NSAF)
Nitric oxide reductase gene cluster					
OHM77_13080	1320		Cytochrome C oxidase subunit I	85.59	4.19E-06
OHM77_13075	594		SCO1/SenC family protein	96.51	2.33E-04
OHM77_13070	426		DUF1318 domain-containing protein	72.26	2.48E-04
OHM77_13065	630		Putative transcriptional regulatory protein	67.86	
OHM77_13060	435	norC	Nitric oxide reductase subunit C	84.90	7.66E-05
OHM77_13055	1374	norB	Nitric oxide reductase subunit B	101.46	4.02E-06
OHM77_13050	606		Cytochrome C oxidase subunit III	76.57	
OHM77_13045	261		Putative cytochrome C oxidase subunit IV family protein	67.89	
OHM77_13040	780	norQ	Nitric oxide reductase regulatory protein	68.52	2.13E-05
OHM77_13035	984		4Fe-4S binding protein	67.09	
OHM77_13030	1845	norD	Nitric oxide reductase activation protein	70.68	
OHM77_13025	393		Putative SirB family protein YchQ	89.00	
OHM77_13020	720		Crp/Fnr family transcriptional regulator	98.14	7.69E-05
Nitrous oxide reductase gene cluster					
OHM77_08590	474	nosL	Putative nitrous oxide reductase accessory protein NosL	128.25	
OHM77_08595	627	nosL	Putative nitrous oxide reductase accessory protein NosL	94.11	4.68E-04
OHM77_08600	159		hypothetical protein	35.16	
OHM77_08605	3966		hypothetical protein	103.60	3.21E-04
OHM77_08610	1923		hypothetical protein	76.72	3.02E-05
OHM77_05915	189		hypothetical protein	37.73	
OHM77_05920	444		hypothetical protein	28.99	9.38E-05
OHM77_05925	774		hypothetical protein	90.30	7.15E-05
OHM77_05930	735		hypothetical protein	95.44	9.80E-05
OHM77_05935	438		hypothetical protein	47.42	
OHM77_05940	3150		hypothetical protein	76.06	1.02E-04
OHM77_05945	861		Pantothenate synthetase	64.99	4.91E-05
OHM77_05950	393		Aspartate 1-decarboxylase	99.10	4.95E-04
OHM77_05955	1827		hypothetical protein	116.65	1.47E-04
OHM77_05960	756		hypothetical protein	102.00	
OHM77_05965	1920		hypothetical protein	61.69	7.79E-05
OHM77_05970	1371		Type I secretion system membrane fusion protein PrsE	63.67	2.32E-04
OHM77_05975	2190		Leukotoxin export ATP-binding protein LtxB	91.22	1.05E-04
OHM77_05980	1620		Putative nitrite reductase	80.01	2.55E-04
OHM77_05985	1146		PqqA peptide cyclase	89.05	5.07E-05
OHM77_05990	501		Thiol-disulfide oxidoreductase ResA	53.07	4.93E-04
OHM77_05995	828	nosY	Putative ABC transporter permease protein	101.38	
OHM77_06000	468		hypothetical protein	64.85	2.91E-04
OHM77_06005	858	nosF	Putative ABC transporter ATP-binding protein nosF	81.07	4.39E-04
OHM77_06010	978		Ferredoxin-type protein NapH family	110.47	1.07E-04
OHM77_06015	867		4Fe-4S dicluster domain-containing protein	77.57	6.70E-04
OHM77_06020	1395	nosD	Nitrous oxide reductase family maturation protein	98.68	1.58E-04
OHM77_06025	978		hypothetical protein	101.89	3.28E-04
OHM77_06030	738		hypothetical protein	129.95	2.45E-03
OHM77_06035	306		Cytochrome C-like domain protein	103.20	1.94E-03
OHM77_06040	2301	nosZ	Nitrous-oxide reductase	302.18	1.19E-02
OHM77_06045	675		Response regulator transcription factor	78.77	1.56E-04
OHM77_06050	1449		hypothetical protein	87.78	6.51E-05
OHM77_06055	2301	nosZ	Nitrous-oxide reductase	289.38	1.19E-02
OHM77_06060	489		hypothetical protein	84.30	1.82E-04
OHM77_06065	513		hypothetical protein	85.26	2.33E-03
Nitrate and nitrite reductase and transport gene clusters					
OHM77_06100	477	nirH	Putative siroheme decarboxylase NirH subunit	44.89	8.73E-05
OHM77_06105	471	nirG	Putative siroheme decarboxylase NirG subunit	77.41	1.12E-04
OHM77_06110	993	nirDL	Putative siroheme decarboxylase NirDL subunit	65.32	4.18E-05
OHM77_06115	1152	nirF	Heme d1 biosynthesis associated protein	66.80	5.95E-04
OHM77_06120	336	nirC	C-type cytochrome	59.86	5.80E-05
OHM77_06125	339		Amicyanin/pseudoazurin family protein	86.56	5.66E-04
OHM77_06130	897		Cytochrome c-552	84.07	2.55E-03
OHM77_06135	1689	nirS	Nitrite reductase	317.67	7.93E-03
OHM77_04910	690	narI	Respiratory nitrate reductase 1 gamma chain	87.86	
OHM77_04915	642	narJ	Respiratory nitrate reductase molybdenum cofactor assembly chaperone	47.66	
OHM77_04920	1548	narH	Respiratory nitrate reductase 1 beta chain	130.45	4.82E-05
OHM77_04925	3753	narG	Respiratory nitrate reductase 1 alpha chain	99.82	6.25E-05
OHM77_04930	1647	narK	Nitrate/nitrite transporter	88.31	
OHM77_04935	1269	narK	Putative nitrate/nitrite transporter	82.37	
OHM77_04940	657	narL	Putative nitrate/nitrite sensor protein NarL	90.73	2.66E-04
OHM77_04945	1926	narX	Nitrate/nitrite sensor protein NarX	75.04	6.47E-05
OHM77_08210	651	narL	Nitrate/nitrite response regulator protein NarL	109.11	
OHM77_08205	1866	narX	Nitrate/nitrite sensor protein NarX	79.95	4.59E-05
OHM77_08200	1728	nirS	Nitrite reductase	102.68	2.12E-04
OHM77_07755	309		Cytochrome c-551	104.57	9.81E-04
OHM77_07750	249	napD	Chaperone NapD	84.84	1.01E-04
OHM77_07745	2550	napA	Periplasmic nitrate reductase large subunit	88.82	2.68E-03
OHM77_07740	861	napG	Ferredoxin-type protein NapG	50.49	6.75E-05
OHM77_07735	894	napH	Ferredoxin-type protein NapH	93.12	2.17E-05
OHM77_07730	456	napB	Periplasmic nitrate reductase electron transfer subunit	58.20	1.26E-03
OHM77_07725	174	napF	Ferredoxin-type protein NapF	31.00	4.84E-05

OHM77_07720	582		NapC/NirT family cytochrome c-type protein	108.63	8.62E-04
OHM77_09985	2436		Assimilatory nitrite reductase large subunit	66.58	
OHM77_09990	348		Assimilatory nitrite reductase small subunit	87.30	
Nitrogenase gene cluster					
OHM77_01845	1119	nifV	Homocitrate synthase	70.96	
OHM77_01850	324	nifW	Nitrogenase-stabilizing/protective protein NifW	70.45	
OHM77_01855	522	nifZ	Nitrogenase molybdenum-iron maturation protein	69.57	
OHM77_01860	282		hypothetical protein	46.92	
OHM77_01865	636		Ion-translocating oxidoreductase complex subunit E	86.39	
OHM77_01870	618		Ion-translocating oxidoreductase complex subunit G	65.42	
OHM77_01875	1092		Ion-translocating oxidoreductase complex subunit D	61.33	
OHM77_01880	1473		Ion-translocating oxidoreductase complex subunit C	43.34	
OHM77_01885	528		Ion-translocating oxidoreductase complex subunit B	27.38	
OHM77_01890	585		Ion-translocating oxidoreductase complex subunit A	74.31	
OHM77_01895	741	nifL	Nitrogen fixation regulatory protein	35.67	
OHM77_01910	1974		hypothetical protein	74.92	7.70E-05
OHM77_01915	1461	nifB	Nitrogenase molybdenum-iron cofactor biosynthesis protein	48.12	
OHM77_01920	888		ADP-ribosyl-[dinitrogen reductase] glycohydrolase	67.14	
OHM77_01925	558		putative protein YaeQ	54.83	1.49E-05
OHM77_01930	444		hypothetical protein	98.36	
OHM77_01935	606		hypothetical protein	66.66	6.86E-05
OHM77_01940	885		NAD()--dinitrogen-reductase ADP-D-ribosyltransferase	66.91	1.25E-05
OHM77_01945	891	nifH	Nitrogenase iron protein	53.49	
OHM77_01950	1449	nifD	Nitrogenase molybdenum-iron protein alpha chain	86.51	
OHM77_01955	1914		Carbon monoxide dehydrogenase 1	41.75	
OHM77_01960	1569	nifK	Nitrogenase molybdenum-iron protein beta chain	87.77	
OHM77_01965	198		Ferredoxin-1	87.60	
OHM77_01970	405		Nitrogenase molybdenum-iron cofactor biosynthesis protein	36.37	
OHM77_01975	864		hypothetical protein	57.13	
OHM77_01980	303		mRNA interferase toxin RelE	47.47	1.56E-04
OHM77_01985	246		Antitoxin YafN	47.81	4.43E-04
OHM77_01990	444		Mycothiol acetyltransferase	29.85	6.88E-05
OHM77_01995	1395	nifE	Nitrogenase molybdenum-iron cofactor biosynthesis protein	51.54	
OHM77_02000	1383	nifN	Nitrogenase molybdenum-iron cofactor biosynthesis protein	88.77	
OHM77_02005	459	nifX	Nitrogenase molybdenum-iron cofactor biosynthesis protein	16.25	
OHM77_02010	288		Ferredoxin-3	18.75	
Ammonium import and assimilation genes					
OHM77_09685	1359	amtB	Ammonia channel	86.90	
OHM77_12200	2610	glnE	Bifunctional glutamine synthetase adenylyltransferase/adenylyl-removing enzym	86.21	4.23E-05
OHM77_13190	1623	nadE	Glutamine-dependent NAD(+) synthetase	91.02	2.64E-04
OHM77_10770	1203	amtB	Ammonia channel	92.14	
OHM77_10620	1413		Glutamine synthetase	121.66	1.27E-03
OHM77_11485	1467	gltD	Glutamate synthase, NADH/NADPH, small subunit	66.98	4.69E-04
OHM77_11480	4656	gltB	Glutamate synthase large subunit	76.53	2.39E-04
Sulfur cycling gene clusters					
OHM77_03540	1695	sir	Sulfite reductase	77.75	
OHM77_03545	753	cysH	Thioredoxin-dependent 5'-adenylylsulfate reductase	67.31	
OHM77_03550	483	tusA	Sulfur carrier protein TusA	80.70	1.09E-04
OHM77_09650	1275	sqrD	Sulfide-quinone reductase	108.45	6.01E-04
OHM77_09655	207		hypothetical protein	56.55	1.07E-03
OHM77_09660	1299		Transcriptional regulatory protein	83.50	2.14E-05
OHM77_09665	1953		hypothetical protein	111.82	2.69E-04
OHM77_09670	582	fccA	Putative sulfide dehydrogenase cytochrome c subunit	89.69	6.10E-04
OHM77_09675	1344	fccB	Putative sulfide dehydrogenase flavoprotein subunit	76.06	1.43E-03
OHM77_12565	603	dsrE	Sulfur carrier protein DsrE2	124.70	2.13E-04
OHM77_12560	228	tusA	Sulfur carrier protein TusA	27.63	6.18E-03
OHM77_12555	978	soeC	Putative sulfite dehydrogenase subunit C	76.07	5.94E-05
OHM77_12550	729	soeB	Putative sulfite dehydrogenase subunit B	64.43	3.71E-04
OHM77_12545	2865	soeA	Putative sulfite dehydrogenase subunit A	85.45	1.09E-04
OHM77_12540	402		hypothetical protein	90.24	2.56E-04
OHM77_12535	519		hypothetical protein	96.90	6.41E-05
OHM77_12530	1284	hdrA	Heterodisulfide reductase iron-sulfur subunit A	83.88	9.47E-04
OHM77_12525	2259	hdrA	Heterodisulfide reductase subunit A	75.96	9.82E-04
OHM77_12520	603	hdrC	Heterodisulfide reductase iron-sulfur subunit C	109.61	4.64E-04
OHM77_12515	897	hdrB	Heterodisulfide reductase subunit B	62.73	6.79E-04
OHM77_12510	933		hypothetical protein	97.54	2.25E-04
OHM77_12505	2028	aprA	Adenylylsulfate reductase subunit alpha	118.65	3.65E-03
OHM77_12500	474	aprB	Adenylylsulfate reductase subunit beta	136.11	4.33E-03
OHM77_12495	1272	sat	Sulfate adenylyltransferase	100.99	1.30E-03
OHM77_12490	1443		Beta-barrel assembly-enhancing protease	96.34	1.16E-04
OHM77_12485	495		Cyclic pyranopterin monophosphate synthase	55.44	7.85E-05
OHM77_12480	471	soxY	Thiosulfate oxidation carrier protein SoxY	197.23	7.01E-04
OHM77_12475	312	soxZ	Thiosulfate oxidation carrier complex protein SoxZ	143.38	3.66E-03
OHM77_12835	834	asrB	Putative anaerobic sulfite reductase subunit B	78.43	2.92E-04
OHM77_12830	1038	asrA	Putative anaerobic sulfite reductase subunit A	113.44	2.08E-04
OHM77_12825	1035		hypothetical protein	64.78	3.79E-04
OHM77_12820	432		hypothetical protein	38.72	
OHM77_12815	1983	hdrA	Heterodisulfide reductase iron-sulfur subunit A	75.26	2.88E-04
OHM77_12810	564		NADH-dependent phenylglyoxylate dehydrogenase subunit gamma	60.69	2.95E-04
OHM77_12805	1206		hypothetical protein	107.36	7.52E-04

OHM77_12800	1176		2-oxoglutarate oxidoreductase subunit KorA	81.15	1.02E-03
OHM77_12795	273		4Fe-4S binding protein	40.79	1.63E-04
OHM77_12790	894	hdrB	Heterodisulfide reductase subunit B	86.42	2.10E-04
OHM77_12785	423	hdrC	Heterodisulfide reductase iron-sulfur subunit C	46.70	3.61E-04
OHM77_04440	333		Sulfurtransferase TusE/DsrC/DsvC family	107.47	2.55E-03
OHM77_04445	609		CRISPR-associated endonuclease Cas6	88.36	8.19E-05
OHM77_04450	216		hypothetical protein	39.50	
OHM77_04455	1296	dsrA	Sulfite reductase, dissimilatory-type subunit alpha	91.50	1.95E-03
OHM77_04460	1074	dsrB	Sulfite reductase, dissimilatory-type subunit beta	110.43	1.13E-03
OHM77_04465	393		Sulfurtransferase DsrE/TusD	90.40	9.97E-04
OHM77_04470	366		Intracellular sulfur oxidation protein DsrF/TusC	65.53	6.38E-04
OHM77_04475	297		Protein DsrH/TusB	101.48	3.38E-04
OHM77_04480	336		Sulfurtransferase DsrC/TusE	84.88	4.97E-04
OHM77_05725	642	napC	Cytochrome c-type protein NapC	113.70	1.60E-04
OHM77_05730	909		Cytochrome c-552	87.98	1.84E-03
OHM77_05735	582	fccA	Putative sulfide dehydrogenase cytochrome c subunit	154.79	4.48E-03
OHM77_05740	1272	fccB	Putative sulfide dehydrogenase flavoprotein subunit	217.98	9.49E-03
OHM77_02120	1698		Putative bifunctional SAT/APS kinase	81.83	4.39E-05
Formate and hydrogen oxidation gene clusters					
OHM77_08935	1425	hydB	Periplasmic [NiFe]-hydrogenase large subunit	61.39	2.91E-05
OHM77_08940	1011	hydA	Periplasmic [NiFe]-hydrogenase small subunit	57.98	4.65E-05
OHM77_08945	1449		Hydrogenase transcriptional regulatory protein	62.62	1.22E-04
OHM77_08950	1743		[NiFe]-hydrogenase maturation factor, HypX/HoxX type	48.28	5.86E-05
OHM77_08955	1050	hypE	Carbamoyl dehydratase HypE	43.22	2.63E-05
OHM77_08960	1146	hypD	Hydrogenase maturation factor HypD	61.47	9.65E-06
OHM77_08965	237		Hydrogenase maturation factor HupF/HypC	38.07	
OHM77_08970	2286	hypF	Carbamoyltransferase HypF	51.00	1.57E-05
OHM77_08975	807	hypB	Hydrogenase maturation factor HypB	70.94	4.12E-05
OHM77_08980	342		Hydrogenase maturation factor HypA/HybF	73.10	4.07E-05
OHM77_08985	1101		Niquel-dependent hydrogenase large subunit	49.62	3.01E-05
OHM77_08990	552	hybE	[NiFe]-hydrogenase assembly chaperone HybE	44.44	
OHM77_08995	246		Hydrogenase maturation factor HupF/HypC	62.04	
OHM77_09000	498	hybD	Hydrogenase maturation protease	57.82	1.62E-04
OHM77_09005	1701		Niquel-dependent hydrogenase large subunit	83.58	1.12E-03
OHM77_09010	1158	hybB	Niquel-dependent hydrogenase cytochrome subunit	55.83	
OHM77_09015	978	hybA	Hydrogenase operon protein	41.55	7.64E-04
OHM77_09020	1140		Niquel-dependent hydrogenase small subunit	83.58	1.11E-03
OHM77_12300	525	hycG	Formate hydrogenlyase subunit 7	42.33	4.23E-05
OHM77_12295	1569	hycE	Formate hydrogenlyase subunit 5	69.71	8.98E-05
OHM77_12290	1452	hycB	NADH-quinone oxidoreductase subunit 2	94.16	
OHM77_12285	669		Hydrogenase 4 component E	70.29	
OHM77_12280	948	hycD	Formate hydrogenlyase subunit 4	61.74	
OHM77_12275	2016	hycC	Hydrogenase 4 component B	74.12	8.22E-06
OHM77_05360	1824	hoxF	Putative NAD-reducing hydrogenase HoxS subunit alpha	39.97	4.09E-05
OHM77_05365	690	hoxU	Putative NAD-reducing hydrogenase HoxS subunit gamma	83.21	2.37E-04
OHM77_05370	540	hoxY	Putative NAD-reducing hydrogenase HoxS subunit delta	82.84	2.57E-05
OHM77_05375	1461	hoxH	Putative NAD-reducing hydrogenase HoxS subunit beta	82.80	9.08E-05
Calvin-Benson-Bassham cycle genes					
OHM77_12185	684		Ribulose-phosphate 3-epimerase	66.37	3.04E-04
OHM77_06505	1065		Fructose-bisphosphate aldolase	82.83	8.70E-04
OHM77_06515	1182		Phosphoglycerate kinase	60.22	2.53E-04
OHM77_06520	999		Glyceraldehyde-3-phosphate dehydrogenase	133.66	1.24E-03
OHM77_06525	2019		Transketolase	94.04	4.72E-04
OHM77_06530	876		Phosphoribulokinase	98.80	2.15E-04
OHM77_06535	1371		Ribulose 1,5-bisphosphate carboxylase	82.35	2.92E-04
OHM77_06585	672		Ribose-5-phosphate isomerase	96.92	1.81E-04
OHM77_13365	747		Triosephosphate isomerase	76.24	9.97E-04
OHM77_05020	1272		Pyrophosphate:fructose 6-phosphate 1-phosphotransferase	70.85	3.61E-04
NADH dehydrogenase (complex I) and succinate dehydrogenase (complex II) gene clusters					
OHM77_13445	1497	nuoN	NADH-quinone oxidoreductase subunit N	67.19	
OHM77_13440	1476	nuoM	NADH-quinone oxidoreductase subunit M	88.10	
OHM77_13435	2013	nuoL	NADH-quinone oxidoreductase subunit L	80.81	3.84E-05
OHM77_13430	306	nuoK	NADH-quinone oxidoreductase subunit K	113.79	
OHM77_13425	609	nuoJ	NADH-quinone oxidoreductase subunit J	75.36	
OHM77_13420	498	nuoI	NADH-quinone oxidoreductase subunit I	51.21	6.18E-04
OHM77_13415	1062	nuoH	NADH-quinone oxidoreductase subunit H	47.80	7.86E-06
OHM77_13410	1965	nuoG	NADH-quinone oxidoreductase subunit G	78.68	4.72E-04
OHM77_13405	1341	nuoF	NADH-quinone oxidoreductase subunit F	58.29	3.96E-04
OHM77_13400	480	nuoE	NADH-quinone oxidoreductase subunit E	75.36	1.45E-04
OHM77_13395	1254	nuoD	NADH-quinone oxidoreductase subunit D	79.31	6.94E-04
OHM77_13390	603	nuoC	NADH-quinone oxidoreductase subunit C	49.60	8.40E-04
OHM77_13385	480	nuoB	NADH-quinone oxidoreductase subunit B	59.90	1.85E-04
OHM77_13380	375	nuoA	NADH-quinone oxidoreductase subunit A	89.24	
OHM77_04225	717	sdhB	Succinate dehydrogenase iron-sulfur subunit	117.57	2.70E-05
OHM77_04230	1788	sdhA	Succinate dehydrogenase flavoprotein subunit	61.19	1.67E-04
OHM77_04235	348	sdhD	Succinate dehydrogenase hydrophobic membrane anchor subunit	57.34	
OHM77_04240	384	sdhC	Succinate dehydrogenase cytochrome b556 subunit	77.27	
Cytochrome c oxidase (complex IV) gene clusters					
OHM77_13135	540	coxB	Cytochrome c oxidase subunit 2	166.11	2.92E-03
OHM77_13130	1701	coxA	Cytochrome c oxidase subunit 1	147.98	5.57E-04

OHM77_13125	900		Heme O synthase	63.13	
OHM77_12980	960	ccoP	Cbb3-type cytochrome c oxidase subunit III	65.89	4.93E-04
OHM77_12975	192	ccoQ	Cbb3-type cytochrome c oxidase subunit IV	57.96	
OHM77_12970	612	ccoO	Cbb3-type cytochrome c oxidase subunit II	93.33	1.08E-03
OHM77_12965	1422	ccoN	Cbb3-type cytochrome c oxidase subunit I	67.31	6.80E-05
ATPase (complex V) gene cluster					
OHM77_09280	771		ATP synthase subunit a	66.47	
OHM77_09285	264		ATP synthase subunit c	62.61	5.28E-05
OHM77_09290	471		ATP synthase subunit b	18.17	1.40E-03
OHM77_09295	528		ATP synthase subunit delta	116.53	2.73E-04
OHM77_09300	1539		ATP synthase subunit alpha	93.54	1.20E-03
OHM77_09305	888		ATP synthase subunit gamma	47.69	3.86E-04
OHM77_09310	1401		ATP synthase subunit beta	102.05	9.47E-04
OHM77_09315	426		ATP synthase subunit epsilon	38.53	3.85E-04
Oxidative and nitrosative stress-related genes					
OHM77_02260	537	msrA	Peptide methionine sulfoxide reductase MsrA	71.50	7.75E-05
OHM77_02265	513	msrB	Peptide methionine sulfoxide reductase MsrB	51.89	1.24E-04
OHM77_02805	441		Universal stress protein	63.03	7.56E-05
OHM77_02815	447		Universal stress protein	53.93	1.86E-05
OHM77_02825	918		Universal stress protein	66.87	8.83E-04
OHM77_02900	873		Universal stress protein	50.29	8.59E-04
OHM77_09195	915	oxyR	Hydrogen peroxide-inducible genes activator	79.41	8.16E-05
OHM77_11845	426		Universal stress protein	71.60	8.08E-04
OHM77_06575	270		Oxidative damage protection protein	83.71	4.23E-04
OHM77_13170	426		Universal stress protein	22.89	
OHM77_13005	522		Putative iron-sulfur cluster repair protein	113.51	5.75E-04
OHM77_13000	444		Universal stress protein	77.80	2.59E-04
OHM77_04885	327		Thioredoxin	140.53	1.62E-03
OHM77_05070	585		Superoxide dismutase	358.22	1.49E-03
OHM77_05320	354		Thioredoxin	21.68	
OHM77_07485	648		Peroxiredoxin	187.48	8.36E-04
OHM77_04195	513		Peroxiredoxin	107.13	4.60E-04
OHM77_00630	1293	hmp	Flavohemoprotein	92.84	5.99E-05
OHM77_00515	918		Universal stress protein	97.82	8.35E-04
OHM77_00180	1278	hmp	Flavohemoprotein	87.83	4.92E-05
OHM77_08495	372		Thioredoxin	97.23	5.53E-04

Table S5: Genes of interest within the genome of *Candidatus* Nitricoxidivorans perseverans and their abundance in the metatranscriptome (RPKM: reads per kilo base per million mapped reads) and metaproteome (NSAF: normalized spectral abundance factor).

Locus_tag	Length (bp)	Gene name	Product (annotation)	Abudance in transcriptome (RPKM)	Abudance in proteome (NSAF)
Nitric oxide reductase gene cluster					
OEL88_02975	651		Transcriptional regulatory protein	115.24	
OEL88_02980	1389		Transcriptional regulatory protein	117.28	
OEL88_02985	438	norC	Nitric oxide reductase subunit C	167.17	
OEL88_02990	1377	norB	Nitric oxide reductase subunit B	161.29	4.01E-06
OEL88_02995	600		Cytochrome C oxidase subunit III	132.83	
OEL88_03000	261		Cytochrome C oxidase subunit IV family protein	125.32	
OEL88_03005	786	norQ	Nitric oxide reductase regulatory protein	138.10	
OEL88_03010	1020		4Fe-4S binding protein	85.42	
OEL88_03015	1881	norD	Nitric oxide reductase activation protein	127.85	1.03E-05
OEL88_03020	705		ABC-type branched-chain amino acid transport systems, ATPase component	80.97	
OEL88_03025	768		ABC-type branched-chain amino acid transport systems, ATPase component	88.17	
OEL88_03030	1059		ABC-type branched-chain amino acid transport systems, permease component	118.41	
OEL88_03035	924		ABC-type branched-chain amino acid transport systems, permease component	71.16	
OEL88_03040	1338		ABC-type branched-chain amino acid transport systems, substrate-binding comp	177.05	
OEL88_03045	306		Cytochrome C	151.29	
Nitrous oxide reductase gene cluster					
OEL88_07000	828	nosY	Putative ABC transporter permease protein	150.60	
OEL88_07005	489	nosL	Nitrous oxide reductase accesory protein	128.18	
OEL88_07010	861	nosF	Putative ABC transporter ATP-binding protein nosF	136.08	
OEL88_07015	981		Ferredoxin-type protein, NapH/MauN family	125.89	2.26E-05
OEL88_07020	867		4Fe-4S domain-containing protein	83.80	
OEL88_07025	1377	nosD	Nitrous oxide reductase family maturation protein	145.25	
OEL88_07030	990		hypothetical protein	171.65	
OEL88_07035	729		Cytochrome C	273.73	5.36E-04
OEL88_07040	309		Cytochrome C	206.26	3.60E-05
OEL88_07045	2298	nosZ	Nitrous-oxide reductase	247.59	2.44E-03
OEL88_07050	654		Response regulator transcription factor	164.62	
OEL88_07055	1146		Sensor histidine kinase	109.39	
OEL88_07060	2670		Sensor histidine kinase	106.64	
OEL88_07065	2295	nosZ	Nitrous-oxide reductase	240.74	2.44E-03
OEL88_07070	537		hypothetical protein	147.04	
OEL88_07075	489		hypothetical protein	109.59	
Nitrate and nitrite reductase and transport gene clusters					
OEL88_00220	1140		Ribosomal protein methyltransferase	82.50	
OEL88_00225	1722	nirS	Nitrite reductase	124.31	2.96E-04
OEL88_00230	618		hypothetical protein	101.47	
OEL88_00235	291		hypothetical protein	107.70	
OEL88_03805	309		Cytochrome c-551	141.60	
OEL88_03810	279	napD	Nitrate reductase biosynthesis chaperone NapD	120.52	
OEL88_03815	2541	napA	Periplasmic nitrate reductase large subunit	242.15	7.60E-04
OEL88_03820	888	napG	Ferredoxin-type protein NapG	132.42	
OEL88_03825	894	napH	Ferredoxin-type protein NapH	162.03	
OEL88_03830	453	napB	Periplasmic nitrate reductase electron transfer subunit	182.77	1.84E-04
OEL88_03835	471	napF	Ferredoxin-type protein NapF	112.02	
OEL88_03840	588		NapC/NirT family cytochrome c-type protein	246.13	6.63E-04
OEL88_07100	1890	kup	Probable potassium transport system protein	112.84	
OEL88_07105	492	nirH	Putative siroheme decarboxylase NirH subunit	158.43	
OEL88_07110	465	nirG	Putative siroheme decarboxylase NirG subunit	89.13	
OEL88_07115	981	nirDL	Putative siroheme decarboxylase NirDL subunit	144.43	
OEL88_07120	1170	nirF	Heme d1 biosynthesis associated protein	119.52	6.16E-05
OEL88_07125	309	nirC	C-type cytochrome	111.64	
OEL88_07130	315		Amicyanin/pseudoazurin family protein	143.07	
OEL88_07135	870	nirB	Cytochrome c-552	278.09	2.39E-04
OEL88_07140	1704	nirS	Nitrite reductase	677.67	7.57E-04
OEL88_08585	612		hypothetical protein	167.79	
OEL88_08590	2718		Molybdopterin oxidoreductase family protein	89.60	
OEL88_08595	2985		hypothetical protein	107.42	
OEL88_08600	1842		hypothetical protein	103.58	
OEL88_08605	537		hypothetical protein	123.84	
OEL88_08610	339		hypothetical protein	83.89	
OEL88_08615	330		Assimilatory nitrite reductase small subunit	127.92	
OEL88_08620	2448		Assimilatory nitrite reductase large subunit	136.96	
OEL88_08625	795	nrtD	Nitrate/nitrite transport system, ATP-binding protein	135.14	
OEL88_08630	915	nrtB	Nitrate/nitrite transport system permease	148.58	
OEL88_08635	1242	nrtA	Nitrate/nitrite transporter, substrate-binding protein	138.69	
Nitrogenase gene cluster					
OEL88_10025	900	nifH	Nitrogenase iron protein	107.14	
OEL88_10030	1473	nifD	Nitrogenase molybdenum-iron protein alpha chain	138.04	
OEL88_10035	1569	nifK	Nitrogenase molybdenum-iron protein beta chain	140.77	
OEL88_10040	222		Protein nifT	75.19	
OEL88_10045	198		Ferredoxin-1	109.54	
OEL88_10050	717		Nitrogenase iron-molybdenum cofactor biosynthesis protein	86.06	
OEL88_10055	279		hypothetical protein	76.94	
OEL88_10060	981		hypothetical protein	108.70	
OEL88_10065	309		hypothetical protein	54.08	
OEL88_10070	270		hypothetical protein	71.23	
OEL88_10075	450		Mycothiol acetyltransferase	119.51	

OEL88_10080	1068		Molybdenum import ATP-binding protein	72.59	
OEL88_10085	672		Molybdenum transport system permease protein ModB	124.49	
OEL88_10090	825		Molybdenum transport system permease protein	98.20	
OEL88_10095	744		Molybdate-binding protein ModA	114.02	
OEL88_10100	810		Molybdenum transport protein	118.26	
OEL88_10105	435		hypothetical protein	126.60	
OEL88_10110	546		hypothetical protein	194.91	
OEL88_10115	2664		hypothetical protein	116.33	
OEL88_10120	1632		hypothetical protein	97.78	
OEL88_10125	1425	nifE	Nitrogenase molybdenum-iron cofactor biosynthesis protein	119.88	
OEL88_10135	1383	nifN	Nitrogenase molybdenum-iron cofactor biosynthesis protein	108.21	
OEL88_10140	411	nifX	Nitrogenase molybdenum-iron cofactor biosynthesis protein	88.10	
OEL88_10145	438		hypothetical protein	91.36	
OEL88_10150	474		hypothetical protein	98.37	
OEL88_10155	210		hypothetical protein	74.46	
OEL88_15240	324	nifW	Nitrogenase-stabilizing/protective protein NifW	134.03	
OEL88_15245	474	nifZ	Nitrogenase molybdenum-iron maturation protein	120.14	
OEL88_15250	840		Foldase protein PrsA	107.41	
OEL88_15255	777		hypothetical protein	95.61	
OEL88_15260	279		hypothetical protein	81.62	
OEL88_15265	672		Ion-translocating oxidoreductase complex subunit E	119.33	
OEL88_15270	603		Ion-translocating oxidoreductase complex subunit G	104.72	
OEL88_15275	1053		Ion-translocating oxidoreductase complex subunit D	148.05	
OEL88_15280	1509		Ion-translocating oxidoreductase complex subunit C	95.32	
OEL88_15285	528		Ion-translocating oxidoreductase complex subunit B	78.77	
OEL88_15290	582		Ion-translocating oxidoreductase complex subunit A	155.28	
OEL88_15295	1548	nifL	Nitrogen fixation regulatory protein	88.95	
OEL88_15300	1560	nifA	Nif-specific regulatory protein	91.51	
OEL88_15305	1248		hypothetical protein	155.06	
OEL88_15310	1179		Adaptive-response sensory-kinase SasA	105.14	
OEL88_15315	1143		hypothetical protein	102.91	
OEL88_15320	1512	nifB	Nitrogenase molybdenum-iron cofactor biosynthesis protein	139.49	
OEL88_15325	291		hypothetical protein	140.58	
OEL88_15330	468		hypothetical protein	74.82	
Ammonium import and assimilation genes					
OEL88_00980	1473	gltD	Glutamate synthase, NADH/NADPH, small subunit	111.66	5.07E-05
OEL88_00985	4653	gltB	Glutamate synthase large subunit	156.64	2.91E-05
OEL88_00990	1341		NADP-specific glutamate dehydrogenase	137.73	2.06E-06
OEL88_04810	1479	amtB	Ammonia channel	122.56	
OEL88_12025	2661	glnE	Bifunctional glutamine synthetase adenylyltransferase/adenylyl-removing enzym	126.35	7.26E-06
OEL88_13395	1206	amtB	Ammonia channel	129.54	
OEL88_13485	1410		Glutamine synthetase	192.40	2.45E-04
Sulfur cycling gene clusters					
OEL88_00440	945	cysB	HTH-type transcriptional regulator CysB	161.33	
OEL88_00445	780		Probable membrane transporter protein	113.68	
OEL88_00450	1704	sir	Putative sulfite reductase	125.49	
OEL88_00455	519		hypothetical protein	168.81	
OEL88_00460	717	cysH	Thioredoxin-dependent 5'-adenylylsulfate reductase	126.36	
OEL88_00465	921	cysD	Sulfate adenylyltransferase small subunit	157.86	
OEL88_00470	1290	cysN	Sulfate adenylyltransferase large subunit	125.99	
OEL88_03155	1272	sqrD	Sulfide-quinone reductase	174.32	7.61E-05
OEL88_03945	1353		Transcriptional regulatory protein	105.80	
OEL88_03950	1959		hypothetical protein	200.92	6.34E-05
OEL88_03955	570	fccA	Putative sulfide dehydrogenase cytochrome c subunit	150.18	
OEL88_03960	1353	fccB	Putative sulfide dehydrogenase flavoprotein subunit	129.12	5.29E-04
OEL88_05265	1296	dsrA	Sulfite reductase, dissimilatory-type subunit alpha	203.63	1.28E-04
OEL88_05270	1077	dsrB	Sulfite reductase, dissimilatory-type subunit beta	303.76	
OEL88_05275	393		Putative sulfurtransferase DsrE	55.47	
OEL88_05280	363		Intracellular sulfur oxidation protein DsrF/TusC	111.02	
OEL88_05285	297		Protein DsrH/TusB	53.93	
OEL88_05290	333		Sulfurtransferase DsrC/TusE	250.62	
OEL88_05295	723		Putative nitrate reductase NarG	120.31	
OEL88_05300	1533		4Fe-4S ferredoxin-type, iron-sulphur binding domain	114.02	
OEL88_06665	642	napC	Cytochrome c-type protein NapC	173.01	
OEL88_06670	906		Cytochrome c-552	138.27	3.66E-05
OEL88_06675	579	fccA	Putative sulfide dehydrogenase cytochrome c subunit	245.34	8.86E-04
OEL88_06680	1281	fccB	Putative sulfide dehydrogenase flavoprotein subunit	275.23	2.33E-04
OEL88_07715	615	dsrE	Sulfur carrier protein DsrE2	102.12	
OEL88_07720	228	tusA	Sulfur carrier protein TusA	172.61	1.92E-03
OEL88_07725	978	soeC	Putative sulfite dehydrogenase subunit C	159.39	
OEL88_07730	732	soeB	Putative sulfite dehydrogenase subunit B	143.57	
OEL88_07735	2874	soeA	Putative sulfite dehydrogenase subunit A	136.16	4.03E-05
OEL88_07740	405		hypothetical protein	168.69	
OEL88_07745	516		hypothetical protein	89.68	
OEL88_07750	1287	hdrA	Heterodisulfide reductase iron-sulfur subunit A	115.35	
OEL88_07755	2256	hdrA	Heterodisulfide reductase subunit A	163.37	2.67E-04
OEL88_07760	621	hdrC	Heterodisulfide reductase iron-sulfur subunit C	151.54	
OEL88_07765	897	hdrB	Heterodisulfide reductase subunit B	155.43	2.47E-05
OEL88_07770	591		hypothetical protein	103.82	
OEL88_07775	2073		Putative nitric oxide reductase activation protein	91.76	

OEL88_07780	816		Putative nitric oxide reductase regulatory protein	105.50	
OEL88_07785	1632		Acetolactate synthase large subunit	141.20	
OEL88_07790	1590		hypothetical protein	145.45	
OEL88_07795	1329		Phenylacetate-coenzyme A ligase	144.96	
OEL88_07800	1023		UDP-N-acetylglucosamine 4-epimerase	158.56	
OEL88_07805	1296		UDP-N-acetyl-D-glucosamine 6-dehydrogenase	145.33	
OEL88_07810	894		hypothetical protein	184.36	
OEL88_07815	2025	aprA	Adenylylsulfate reductase subunit alpha	219.27	1.58E-03
OEL88_07820	474	aprB	Adenylylsulfate reductase subunit beta	205.91	2.39E-03
OEL88_07825	1206	sat	Sulfate adenylyltransferase	151.53	1.90E-04
OEL88_07830	1446		Beta-barrel assembly-enhancing protease	152.00	
OEL88_07835	450		Response regulator rcp1	172.29	
OEL88_07840	489		Cyclic pyranopterin monophosphate synthase	186.85	
OEL88_07845	474	soxY	Thiosulfate oxidation carrier protein SoxY	213.55	1.76E-05
OEL88_07850	315	soxZ	Thiosulfate oxidation carrier complex protein SoxZ	117.07	1.33E-04
OEL88_07855	546		ECF RNA polymerase sigma factor SigR	127.96	
OEL88_07860	699		hypothetical protein	191.79	
OEL88_07865	870	soxA	Sulfur oxidation c-type cytochrome SoxA	211.80	
OEL88_07870	618	soxX	Sulfur oxidation c-type cytochrome SoxX	129.11	
OEL88_07875	1719	soxB	Sulfur oxidation protein SoxB	169.44	
Formate and hydrogen oxidation gene clusters					
OEL88_05130	1788	hoxF	Putative NAD-reducing hydrogenase HoxS subunit alpha	112.38	1.54E-05
OEL88_05135	705	hoxU	Putative NAD-reducing hydrogenase HoxS subunit gamma	139.55	
OEL88_05140	564	hoxY	Putative NAD-reducing hydrogenase HoxS subunit delta	156.68	
OEL88_05145	1485	hoxH	Putative NAD-reducing hydrogenase HoxS subunit beta	166.94	4.28E-05
OEL88_05150	441		hypothetical protein	77.83	
OEL88_06150	1101		Nickel-dependent hydrogenase, small subunit	110.13	
OEL88_06155	1797		Nickel-dependent hydrogenase, large subunit	160.74	
OEL88_06160	702		Nickel-dependent hydrogenase b-type cytochrome subunit	176.92	
OEL88_06165	522		Hydrogenase maturation protease	81.47	
OEL88_12185	2013	hycC	Hydrogenase 4 component B	128.07	8.20E-06
OEL88_12190	954	hycD	Formate hydrogenlyase subunit 4	155.40	
OEL88_12195	666	hyfE	Hydrogenase 4 membrane component E	139.91	
OEL88_12200	1452	hyfF	Hydrogenase 4 component F	117.07	
OEL88_12205	1557	hycE	Formate hydrogenlyase subunit 5	88.52	
OEL88_12210	519	hycG	Formate hydrogenlyase subunit 7	68.72	
OEL88_12490	1764		Nickel-dependent hydrogenase, large subunit	123.97	
OEL88_12495	1266		hypothetical protein	126.40	
OEL88_12500	660		hypothetical protein	128.92	
OEL88_12505	1092	hoxK	Nickel-dependent hydrogenase, small subunit	109.78	
OEL88_12710	2430	fdnG	Formate dehydrogenase-N major subunit	267.40	5.58E-05
OEL88_12715	888	fdnH	Formate dehydrogenase-N iron-sulfur subunit	194.81	2.18E-05
OEL88_12720	621	fdnI	Formate dehydrogenase-N, cytochrome subunit	138.94	
OEL88_12725	918	fdhE	Formate dehydrogenase, accesory protein FdhE	129.41	
OEL88_16715	1200		Hydrogenase-2 small chain	144.90	6.04E-05
OEL88_16720	987	hybA	Hydrogenase-2 operon protein HybA	173.25	
OEL88_16725	1155	hybB	putative Ni/Fe-hydrogenase 2 b-type cytochrome subunit	138.39	
OEL88_16730	1707		Hydrogenase-2 large chain	138.66	
OEL88_16735	492	hybD	Hydrogenase 2 maturation protease	120.42	
OEL88_16740	261	hypC	Hydrogenase maturation factor	81.04	
OEL88_16745	489		Hydrogenase expression/formation protein	111.17	
OEL88_16750	342	hypA	Hydrogenase maturation factor HypA	122.04	
OEL88_16755	1086	hypB	Hydrogenase maturation factor HypB	168.79	
OEL88_16760	1110	hypF	Carbamoyltransferase HypF	99.12	
OEL88_16765	228	hypC	Hydrogenase maturation factor HypC	174.49	
OEL88_16770	1131	hypD	Hydrogenase maturation factor HypD	110.18	
OEL88_16775	1053	hypE	Carbamoyl dehydratase HypE	125.53	
OEL88_16780	1701	hoxX	Hydrogenase maturation factor hoxX	89.09	
OEL88_16785	1455	hoxA	Hydrogenase transcriptional regulatory protein	106.82	1.71E-05
OEL88_16790	996		Periplasmic [NiFeSe] hydrogenase small subunit	84.41	
OEL88_16795	1443		Periplasmic [NiFeSe] hydrogenase large subunit	84.57	
Calvin-Benson-Bassham cycle genes					
OEL88_05455	714		Triosephosphate isomerase	108.55	1.36E-04
OEL88_11995	693		Ribulose-phosphate 3-epimerase	143.64	
OEL88_14590	1065		Fructose-bisphosphate aldolase	172.13	4.54E-04
OEL88_14600	1182		Phosphoglycerate kinase	130.77	5.61E-05
OEL88_14605	999		Glyceraldehyde-3-phosphate dehydrogenase	232.08	4.76E-04
OEL88_14610	2016		Transketolase	175.05	7.40E-05
OEL88_14615	879		Phosphoribulokinase	152.35	
OEL88_14620	1053		Fructose-1,6-bisphosphatase	189.39	
OEL88_14640	1380		Ribulose 1,5-bisphosphate carboxylase	113.53	
OEL88_14695	672		Ribose-5-phosphate isomerase	129.37	
NADH dehydrogenase (complex I) and succinate dehydrogenase (complex II) gene clusters					
OEL88_05375	1482	nuoN	NADH-quinone oxidoreductase subunit N	112.66	
OEL88_05380	1476	nuoM	NADH-quinone oxidoreductase subunit M	114.14	
OEL88_05385	2037	nuoL	NADH-quinone oxidoreductase subunit L	130.47	1.63E-05
OEL88_05390	315	nuoK	NADH-quinone oxidoreductase subunit K	100.18	
OEL88_05395	624	nuoJ	NADH-quinone oxidoreductase subunit J	147.52	
OEL88_05400	486	nuoI	NADH-quinone oxidoreductase subunit I	161.73	
OEL88_05405	1062	nuoH	NADH-quinone oxidoreductase subunit H	140.47	

OEL88_05410	2124	nuoG	NADH-quinone oxidoreductase subunit G	131.28	2.08E-05
OEL88_05415	1347	nuoF	NADH-quinone oxidoreductase subunit F	108.45	
OEL88_05420	474	nuoE	NADH-quinone oxidoreductase subunit E	139.65	
OEL88_05425	1254	nuoD	NADH-quinone oxidoreductase subunit D	131.95	1.17E-04
OEL88_05430	591	nuoC	NADH-quinone oxidoreductase subunit C	155.77	
OEL88_05435	477	nuoB	NADH-quinone oxidoreductase subunit B	171.82	
OEL88_05440	375	nuoA	NADH-quinone oxidoreductase subunit A	128.19	
OEL88_14275	393	sdhC	Succinate dehydrogenase cytochrome b556 subunit	86.74	
OEL88_14280	339	sdhD	Succinate dehydrogenase hydrophobic membrane anchor subunit	101.61	
OEL88_14285	1788	sdhA	Succinate dehydrogenase flavoprotein subunit	150.15	3.40E-05
OEL88_14290	717	sdhB	Succinate dehydrogenase iron-sulfur subunit	140.21	
Cytochrome bc1 complex (complex III)					
OEL88_12580	711		Cytochrome c1	165.86	
OEL88_12585	1299		Cytochrome b	143.29	
OEL88_12590	597		Ubiquinol-cytochrome c reductase iron-sulfur subunit	167.85	
Cytochrome c oxidase (complex IV) gene clusters					
OEL88_01905	1626	coxA	Cytochrome c oxidase subunit 1	158.58	
OEL88_01910	693	coxB	Cytochrome c oxidase subunit 2	95.17	
OEL88_01920	777	coxC	Cytochrome c oxidase subunit 3	135.21	
OEL88_06870	540	coxB	Cytochrome c oxidase subunit 2	600.73	9.76E-05
OEL88_06875	1698	coxA	Cytochrome c oxidase subunit 1	358.66	
OEL88_06880	894		Heme O synthase	180.65	
OEL88_07410	1014	ccoP	Cbb3-type cytochrome c oxidase subunit III	204.39	1.80E-04
OEL88_07415	183	ccoQ	Cbb3-type cytochrome c oxidase subunit IV	105.46	
OEL88_07420	612	ccoO	Cbb3-type cytochrome c oxidase subunit II	184.37	1.81E-04
OEL88_07425	1422	ccoN	Cbb3-type cytochrome c oxidase subunit I	183.78	
ATPase (complex V) gene cluster					
OEL88_04600	777		ATP synthase subunit a	242.99	
OEL88_04605	264		ATP synthase subunit c	134.68	
OEL88_04610	471		ATP synthase subunit b	67.48	2.95E-04
OEL88_04615	537		ATP synthase subunit delta	166.85	
OEL88_04620	1539		ATP synthase subunit alpha	158.27	4.49E-04
OEL88_04625	885		ATP synthase subunit gamma	157.75	9.38E-05
OEL88_04630	1401		ATP synthase subunit beta	162.24	2.76E-04
OEL88_04635	426		ATP synthase subunit epsilon	92.99	3.91E-05
Oxidative and nitrosative stress-related genes					
OEL88_01370	600	ahpC	Alkyl hydroperoxide reductase subunit C	221.14	
OEL88_02570	570	ahpC	Alkyl hydroperoxide reductase subunit C	504.38	
OEL88_02575	1578	ahpF	Alkyl hydroperoxide reductase subunit F	154.17	
OEL88_02620	993	ccpR	Cytochrome c551 peroxidase	155.72	
OEL88_02625	906	oxyR	Hydrogen peroxide-inducible genes activator	113.44	
OEL88_02630	1131	ccpR	Cytochrome c551 peroxidase	141.83	
OEL88_02855	363		Thioredoxin 2	86.43	
OEL88_04465	915	oxyR	Hydrogen peroxide-inducible genes activator	123.70	
OEL88_05825	438		Thioredoxin 2	67.37	
OEL88_06095	327		Thioredoxin	221.71	9.53E-04
OEL88_06360	585		Superoxide dismutase	174.92	7.58E-04
OEL88_06945	2169		Catalase-peroxidase	100.58	
OEL88_07260	951		Thioredoxin reductase	122.62	2.30E-04
OEL88_07355	456		Putative iron-sulfur cluster repair protein	107.99	
OEL88_08080	543	msrA	Peptide methionine sulfoxide reductase MsrA	255.07	
OEL88_10855	381		Thioredoxin	88.26	
OEL88_14125	501		Thiol peroxidase	131.78	

Table S6: Genes of interest within the genome of *Candidatus* Nitricoxidireducens bremensis and their abundance in the metatranscriptome (RPKM: reads per kilo base per million mapped reads) and metaproteome (NSAF: normalized spectral abundance factor).

	Nitrate (A)	Nitrate (B)	Nitrite (A)	Nitrite (B)	Nitric oxide (A)	Nitric oxide (B)	Nitrous oxide (A)	Nitrous oxide (B)
**Michaelis-Menten**								
**Best-fit values**								
Vmax	4.59	4.48	9.38	9.16	1.44	1.47	2.33	2.22
Km	0.88	0.79	0.55	0.49	0.18	0.15	0.95	1.05
**95% CI (profile likelihood)**								
Vmax	3.80 to 5.47	3.24 to 5.90	7.83 to 11.11	8.17 to 10.20	1.20 to 1.77	1.17 to 1.90	2.13 to 2.54	1.96 to 2.49
Km	0.29 to 2.20	0.02 to 2.99	0.11 to 1.53	0.24 to 0.99	0.08 to 0.37	0.05 to 0.37	0.70 to 1.30	0.69 to 1.62
**Goodness of Fit**								
Degrees of Freedom	9	8	9	9	12	11	14	13
R squared	0.6919	0.4224	0.7669	0.8679	0.7428	0.6575	0.9408	0.9005
**Number of points**								
# of X values	11	10	11	11	14	13	16	15
# Y values analyzed	11	10	11	11	14	13	16	15

	Nitric oxide (A)	Nitric oxide (B)
**Substrate inhibition**		
**Best-fit values**		
Ki	2.05	1.69
**95% CI (profile likelihood)**		
Ki	0.42 to 6.34	0.22 to 5.65
**Goodness of Fit**		
Degrees of Freedom	15	15
R squared	0.7774	0.7269
**Number of points**		
# of X values	18	18
# Y values analyzed	18	18

Table S7: Goodness of fit measures and confidence intervals (95% CI) for the kinetic constants of the enrichment culture for nitrate, nitrite, nitric oxide and nitrous oxide, calculated with the Monod equation and the Haldane equation (only for nitric oxide) for each of the two replicates (A and B).
