# Supplementary material for: Enrichment and characterization of a nitric oxide-reducing microbial community in a continuous bioreactor
Source: Nat Microbiol. 2023 Jul 10;8(8):1574–86. doi: 10.1038/s41564-023-01425-8 (PMC10390337; doi:10.1038/s41564-023-01425-8)
Supplement: Supplementary file 2 — Reporting Summary [file 41564_2023_1425_MOESM2_ESM.pdf]

nature portfolio

Corresponding author(s): Boran Kartal

Last updated by author(s): Jun 7, 2023

Reporting Summary

Nature Portfolio wishes to improve the reproducibility of the work that we publish. This form provides structure for consistency and transparency in reporting. For further information on Nature Portfolio policies, see our Editorial Policies and the Editorial Policy Checklist.

Statistics

For all statistical analyses, confirm that the following items are present in the figure legend, table legend, main text, or Methods section.

n/a	Confirmed	
☐	☒	The exact sample size (*n*) for each experimental group/condition, given as a discrete number and unit of measurement
☐	☒	A statement on whether measurements were taken from distinct samples or whether the same sample was measured repeatedly
☒	☐	The statistical test(s) used AND whether they are one- or two-sided<br>*Only common tests should be described solely by name; describe more complex techniques in the Methods section.*
☒	☐	A description of all covariates tested
☒	☐	A description of any assumptions or corrections, such as tests of normality and adjustment for multiple comparisons
☒	☐	A full description of the statistical parameters including central tendency (e.g. means) or other basic estimates (e.g. regression coefficient) AND variation (e.g. standard deviation) or associated estimates of uncertainty (e.g. confidence intervals)
☒	☐	For null hypothesis testing, the test statistic (e.g. *F*, *t*, *r*) with confidence intervals, effect sizes, degrees of freedom and *P* value noted<br>*Give P values as exact values whenever suitable.*
☒	☐	For Bayesian analysis, information on the choice of priors and Markov chain Monte Carlo settings
☒	☐	For hierarchical and complex designs, identification of the appropriate level for tests and full reporting of outcomes
☒	☐	Estimates of effect sizes (e.g. Cohen's *d*, Pearson's *r*), indicating how they were calculated

Our web collection on statistics for biologists contains articles on many of the points above.

Software and code

Policy information about availability of computer code

Data collection	mass spectrometry ChromStar 7 (1.1.6.4), ProxiMass 2000 (1.0.17.3), NO measurements CldGui (1.4.0.6), N2O measurements Maestro 1 (1.2.3.5/3.2)
Data analysis	Plots and rate calculations were performed using MATLAB R2019a; different metagenomic and metatranscriptomic analyses were performed using Trimmomatic v 0.39, SPAdes 3.15.3, Maxbin 2.2.7, Metabat v 2.12.1, DAS_Tool v 1.1.1, Canu 1.9, Flye 2.9, CheckM v1.1.2, SortMeRNA v 4.1, Prokka v1.14.5, MAFFT v7.407, hmmbuild (HMMER package, v3.3.2), hmmscan (HMMER package, v3.3.2), GTDB-Tk v1.7.075, MiGA 1.1.2.2, FastANI v1.32 and v1.33, IQ-TREE v 1.6.12, coverM 0.6.1, DIAMOND 2.0.15, Enveomics 0.1.1, iTOL 6.7.5; metaproteome analyses were performed with PaSER v2023, ProLuCID 1.3, DTASelect 2.0, minimap2 2.26, EMBOSS 6.5.0; oligonucleotide probe design was performed using ARB software, package v 6.1, SINA (v1.2.12). Kinetics data anlses GraphPad Prism 9 (version 9. 5. 1., GraphPad Software, LLC)

For manuscripts utilizing custom algorithms or software that are central to the research but not yet described in published literature, software must be made available to editors and reviewers. We strongly encourage code deposition in a community repository (e.g. GitHub). See the Nature Portfolio guidelines for submitting code & software for further information.

Data

Policy information about availability of data

All manuscripts must include a data availability statement. This statement should provide the following information, where applicable:

- Accession codes, unique identifiers, or web links for publicly available datasets
- A description of any restrictions on data availability
- For clinical datasets or third party data, please ensure that the statement adheres to our policy

Raw data from metagenomic and metatranscriptomic analyses as well as all metagenome-assembled genomes generated in this study have been deposited in the NCBI under BioProject number PRJNA849246. The metagenome-assembled genomes of Ca. Nitricoxidivorans perseverans (MAG1) and Ca. Nitricoxidireducens bremensis (MAG5) are deposited, respectively, under BioSample numbers SAMN30388482 and SAMN30388483 and genome accession numbers CP107246 and JAOTRT000000000. Metaproteomics data, including raw data files and ProLuCID search results, have been deposited to the ProteomeXchange Consortium via the PRIDE96 under identifier PXD037586. Databases used: SILVA SSU Ref NR 99 138.1, NR (GenBank 249, NCBI), Pfam 35.0, UniProt release 2022_01, KEGG release 101.0, GTDB release 207, Genomes from Earth's Microbiomes (GEM) 2021, FunGene 7.3

Human research participants

Policy information about studies involving human research participants and Sex and Gender in Research.

Reporting on sex and gender	N/A
Population characteristics	N/A
Recruitment	N/A
Ethics oversight	N/A

Note that full information on the approval of the study protocol must also be provided in the manuscript.

Field-specific reporting

Please select the one below that is the best fit for your research. If you are not sure, read the appropriate sections before making your selection.

☒ Life sciences ☐ Behavioural & social sciences ☐ Ecological, evolutionary & environmental sciences

For a reference copy of the document with all sections, see nature.com/documents/nr-reporting-summary-flat.pdf

Life sciences study design

All studies must disclose on these points even when the disclosure is negative.

Sample size	No statistical methods were used to predetermine sample size as is the standard procedure for enrichment culture experiments. For the bioreactor experiment, more than 1500 samples were analyzed corresponding to each measurement day.
Data exclusions	No data was excluded
Replication	Results of all replicate experiments are displayed in the article, and all replicates have been successful. In the microbial kinetics experiments duplicates were performed, each duplicate contained 10-18 distinct rate measurement experiments. For the net yield measurements, six distinct measurements were performed in triplicate. For the fluorescence in situ hybridization imaging, in triplicate experiments, more than 1000 cells were counted per replicate for quantitative analysis.
Randomization	There are no experimental groups in this study. All measurements and experiments were performed using an enrichment culture in a chemostat. Intrinsically all samples taken from the bioreactor are randomized as each cell has the same statistical probability to be sampled.
Blinding	Blinding was not applicable because the study does not involve animals and/or human research participants.

Reporting for specific materials, systems and methods

We require information from authors about some types of materials, experimental systems and methods used in many studies. Here, indicate whether each material, system or method listed is relevant to your study. If you are not sure if a list item applies to your research, read the appropriate section before selecting a response.

Materials & experimental systems

n/a	Involved in the study
☒	☐ Antibodies
☒	☐ Eukaryotic cell lines
☒	☐ Palaeontology and archaeology
☒	☐ Animals and other organisms
☒	☐ Clinical data
☒	☐ Dual use research of concern

Methods

n/a	Involved in the study
☒	☐ ChIP-seq
☒	☐ Flow cytometry
☒	☐ MRI-based neuroimaging
